# Supplementary material for: Real-time assessment of mitochondrial DNA heteroplasmy dynamics at the single-cell level
Source: EMBO J. 2024 Aug 5;43(22):4. doi: 10.1038/s44318-024-00183-5 (PMC11574196; doi:10.1038/s44318-024-00183-5)
Supplement: Supplementary file 1 — Appendix [file 44318_2024_183_MOESM1_ESM.pdf]

Appendix
Real-time assessment of mitochondrial DNA heteroplasmy dynamics at the single-cell level

Rodaria Roussou et al.

*Corresponding author: osman@bio.lmu.de

Contents

Appendix Figure S1 2
Appendix Figure S2 3
Appendix Figure S3 5
Appendix Figure S4 7
Appendix Figure S5 8
Appendix Figure S6 9
Appendix Figure S7 11
Appendix Figure S8 13
Appendix Figure S9 15
Appendix Table S1 17
Appendix Table S2 18
Appendix Table S3 18

Appendix Figure S1

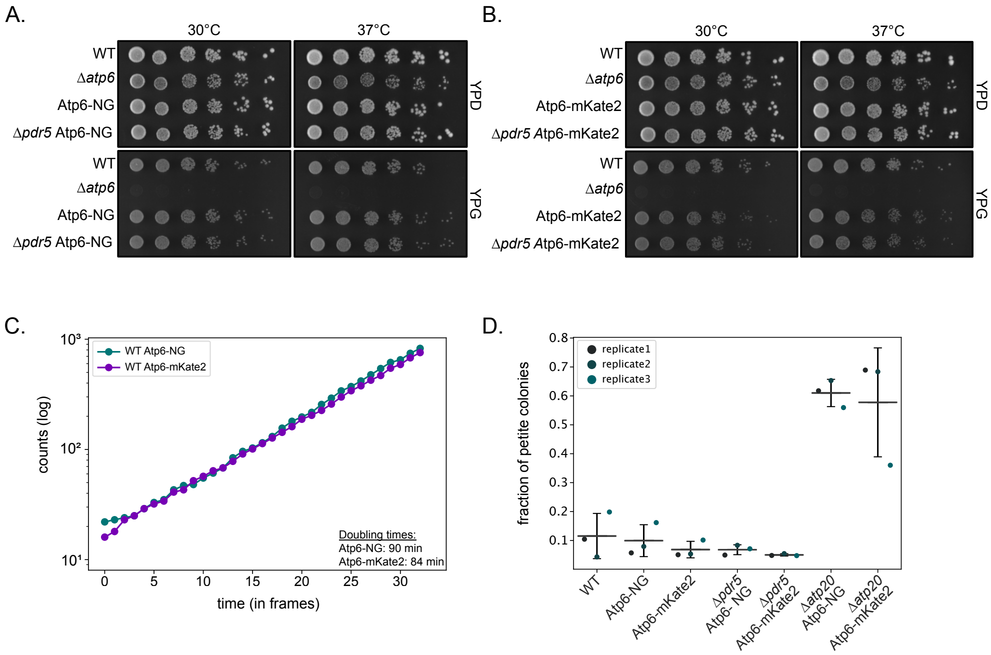

Growth and petite frequency of mtDNA$^{\mathbf{Atp6\text{-}NG}}$ and mtDNA$^{\mathbf{Atp6\text{-}mKate2}}$ strains. (A, B) Drop dilution growth analyses of strains harboring mtDNA$^{\text{Atp6-NG}}$ (A) or mtDNA$^{\text{Atp6-mKate2}}$ (B). Serial dilutions of the indicated strains were spotted on rich medium containing glucose or glycerol as carbon sources and incubated at 30°C or 37°C. **(C)** Growth curves of Atp6-NG and Atp6-mKate2 strains were assessed from timelapse microscopy data of 8-hr recordings (N=6/strain). Cell amounts were derived by segmentation of brightfield images in 15 min intervals. Cell amounts are depicted on the y axis in log scale. **(D)** Petite frequency of indicated strains (n=3). The Δ*atp20* strains, known to have elevated petite levels, were used as controls.

Appendix Figure S2

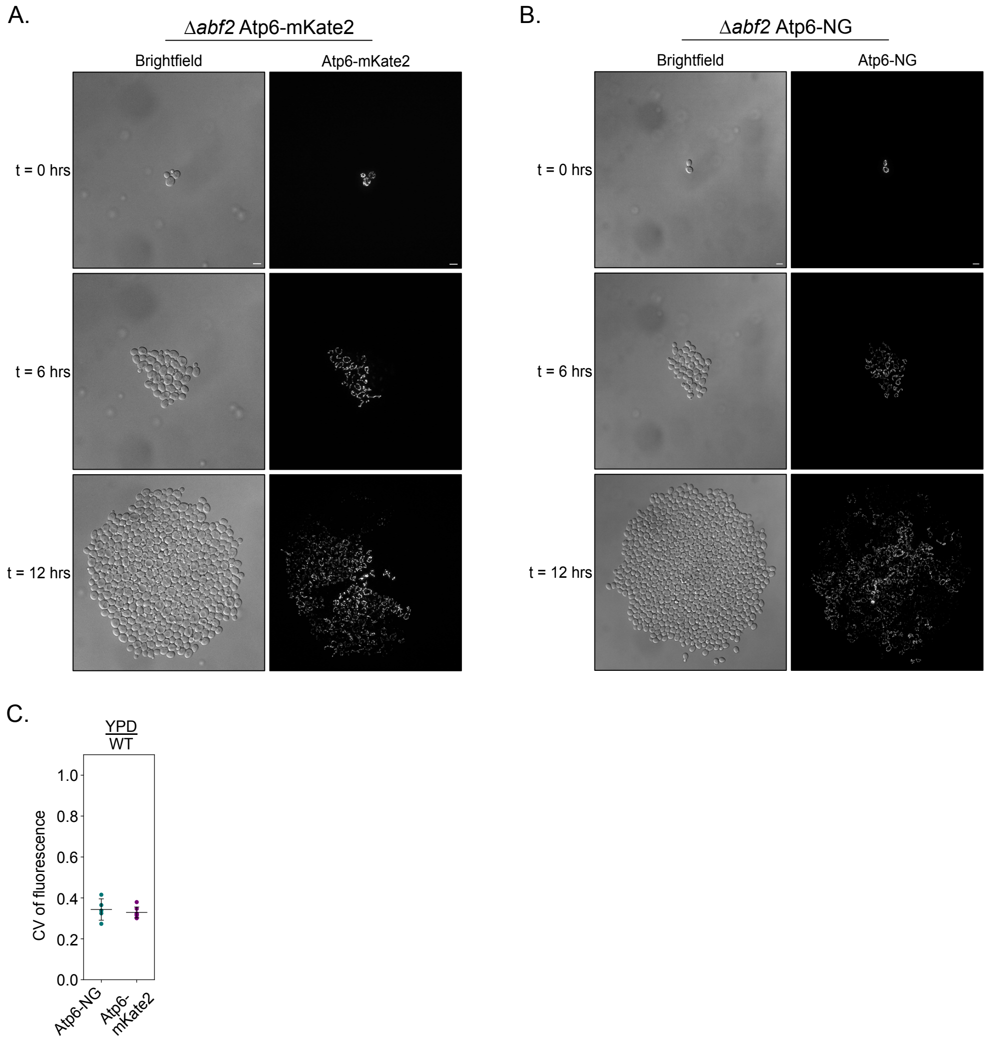

Expression of Atp6-NG and Atp6-mKate2 in Δ*abf2* cells over time. (A, B) Representative images from Δ*abf2* cells expressing (A) Atp6-NG or (B) Atp6-mKate2 (N=3/strain). Cells were imaged for 12 hrs, and three different timepoints are shown (t = 0, 6 and 12 hrs). Cells were first grown in YPG and maintained in minimal media during the microfluidic imaging, to assess mtDNA loss. Fluorescence images are maximum intensity projections

of z-stacks, after deconvolution. Scale bars: 5 μm. **(C)** Coefficient of variation (CV) of fluorescence calculated at the final timepoint of imaging for strains, harboring mtDNA$^{\text{Atp6-NG}}$ or mtDNA$^{\text{Atp6-mKate2}}$ (N=6/strain). Strains were pre-grown in glucose-containing media (YPD).

Appendix Figure S3

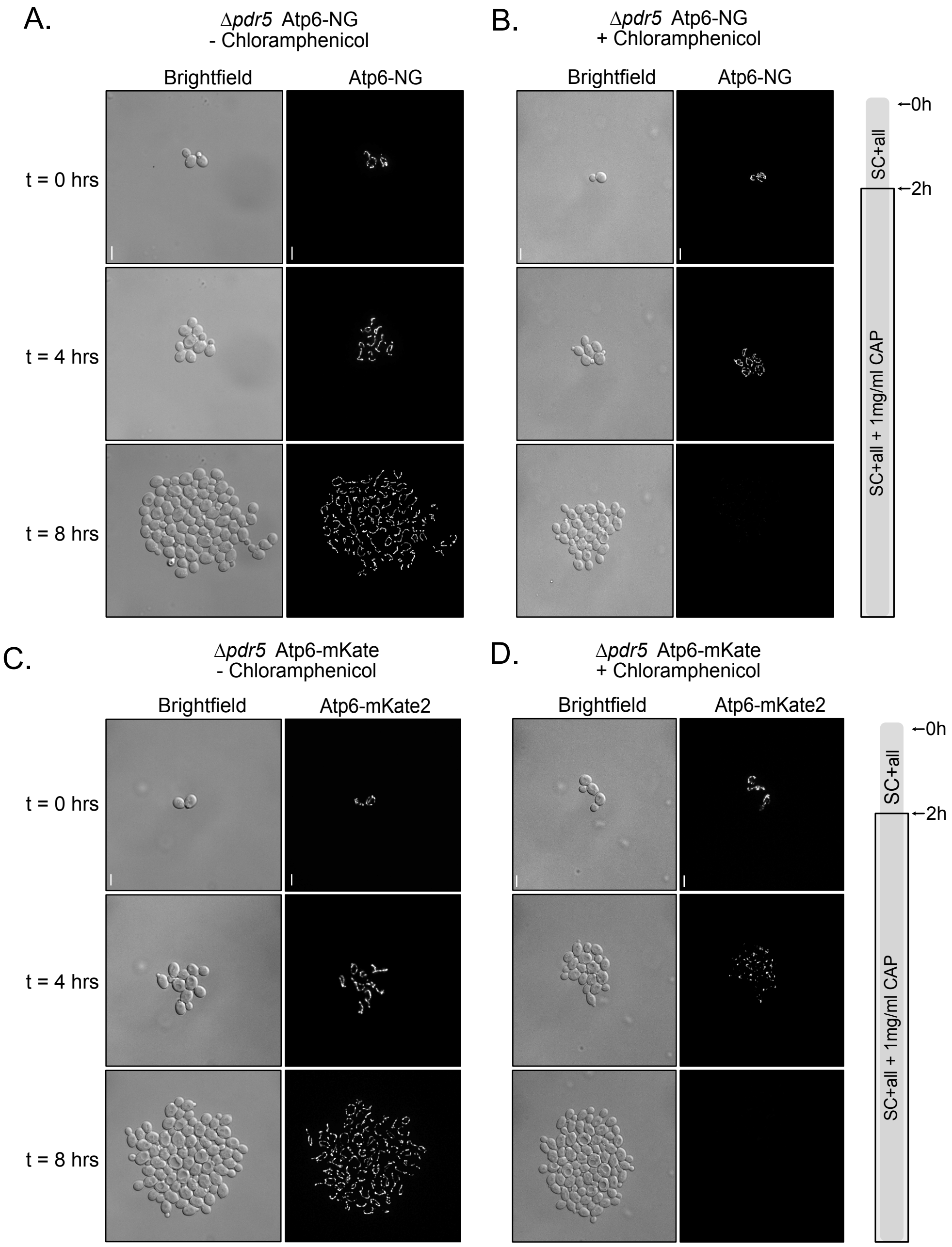

mtDNA$^{\text{Atp6-NG}}$ and mtDNA$^{\text{Atp6-mKate2}}$ strains with and without treatment with Chloramphenicol (CAP). (A, B, C, D) Representative images of Δ*pdr5* cells harboring mtDNA$^{\text{Atp6-NG}}$ (A, B) or mtDNA$^{\text{Atp6-mKate2}}$ (C, D). Cells were imaged for 8 hrs and fresh minimal media was provided throughout each experiment with (B, D) or without (A, C) addition of CAP (1 mg/ml) after the first 2 hrs of recording (N=3/strain). Fluorescence images are maximum intensity projections of z-stacks, after deconvolution. Scale bars: 5 μm.

Appendix Figure S4

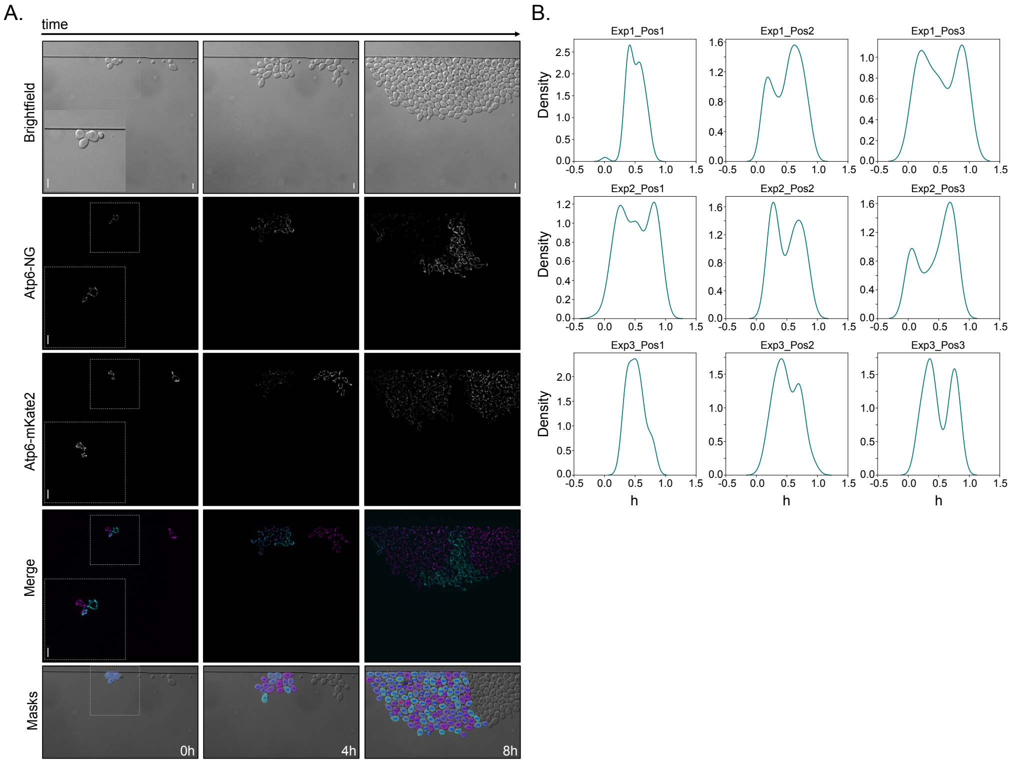

Heteroplasmy distribution amongst individual colonies. (A) The same representative images of Figure 3A, at timepoints 0h, 4h and 8 hrs, without histogram adjustment. Fluorescence images are maximum intensity projections of z-stacks, after deconvolution. Scale bars: 5 μm. **(B)** Heteroplasmy distribution from the last timepoint of each individual diploid population (N=9). An h-value of 0.5 represents a virtually absolute heteroplasmic state. Cells with h-values lower than 0.5 contain a higher proportion of mtDNA$^{\text{Atp6-NG}}$, while cells with h-values above 0.5 contain a higher proportion of mtDNA$^{\text{Atp6-mKate2}}$. Segregation can be observed across all independent populations, with cells distributed across two homoplasmic peaks. Notably, in three cases (Exp1Pos1, Exp3Pos1, Exp3Pos2) segregation is more subtle and the homoplasmic peaks less pronounced.

Appendix Figure S5

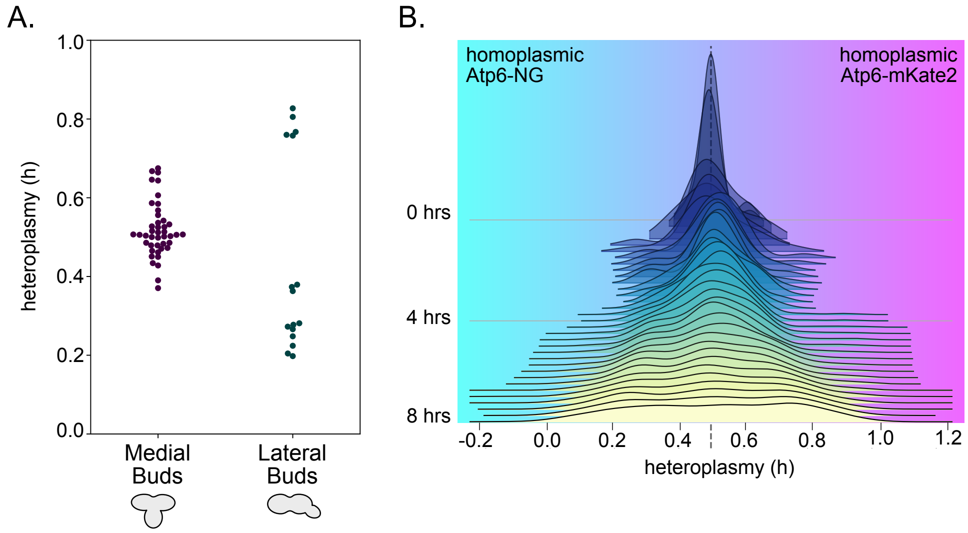

Heteroplasmy levels in medial and lateral buds. (A) Heteroplasmy values for medial and lateral buds from zygotes, assessed upon mating within the first 2hrs of growth in the microfluidic chamber. Each dot represents one single cell. Heteroplasmy (h) values below 0.33 or above 0.71, which are the homoplasmy thresholds, represent cells harboring mainly mtDNA$^{\text{Atp6-NG}}$ or mtDNA$^{\text{Atp6-mKate2}}$, respectively. **(B)** Joyplot of heteroplasmy levels of cells over time, from a total of nine populations, excluding all lateral buds and their progeny. All populations were derived from heteroplasmic zygotes across 8-hr timelapse recordings. The dashed line represents the virtually absolute heteroplasmy value of h=0.5.

Appendix Figure S6

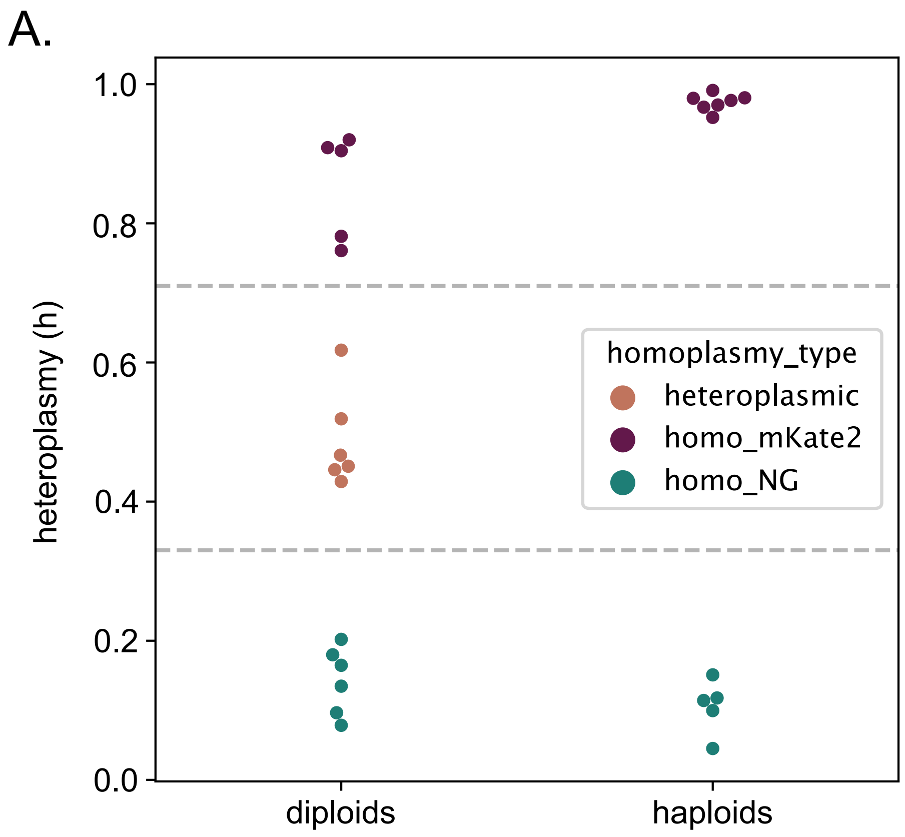

h-values of non-mated haploid cells present in timelapse videos of heteroplasmic diploid cells. (A) During recording of mtDNA heteroplasmy segregation in diploid populations over time, individual haploids cells that did not mate were also present in the same field of view. Diploid cells, classified as homoplasmic for either Atp6-NG or Atp6-mKate2, or still remaining heteroplasmic, were randomly selected from the final timepoint of the microfluidic videos, and their heteroplasmy values were compared to non-mated haploid homoplasmic cells. Application of the heteroplasmy equation places these haploid cells to the extremes of the heteroplasmy values, below 0.33 or above 0.71, comparable to diploid cells characterized as homoplasmic

based on the same homoplasmy thresholds. Each dot represents one single cell. Dashed lines indicate the homoplasmy thresholds, at h=0.33 and 0.71, respectively.

Appendix Figure S7

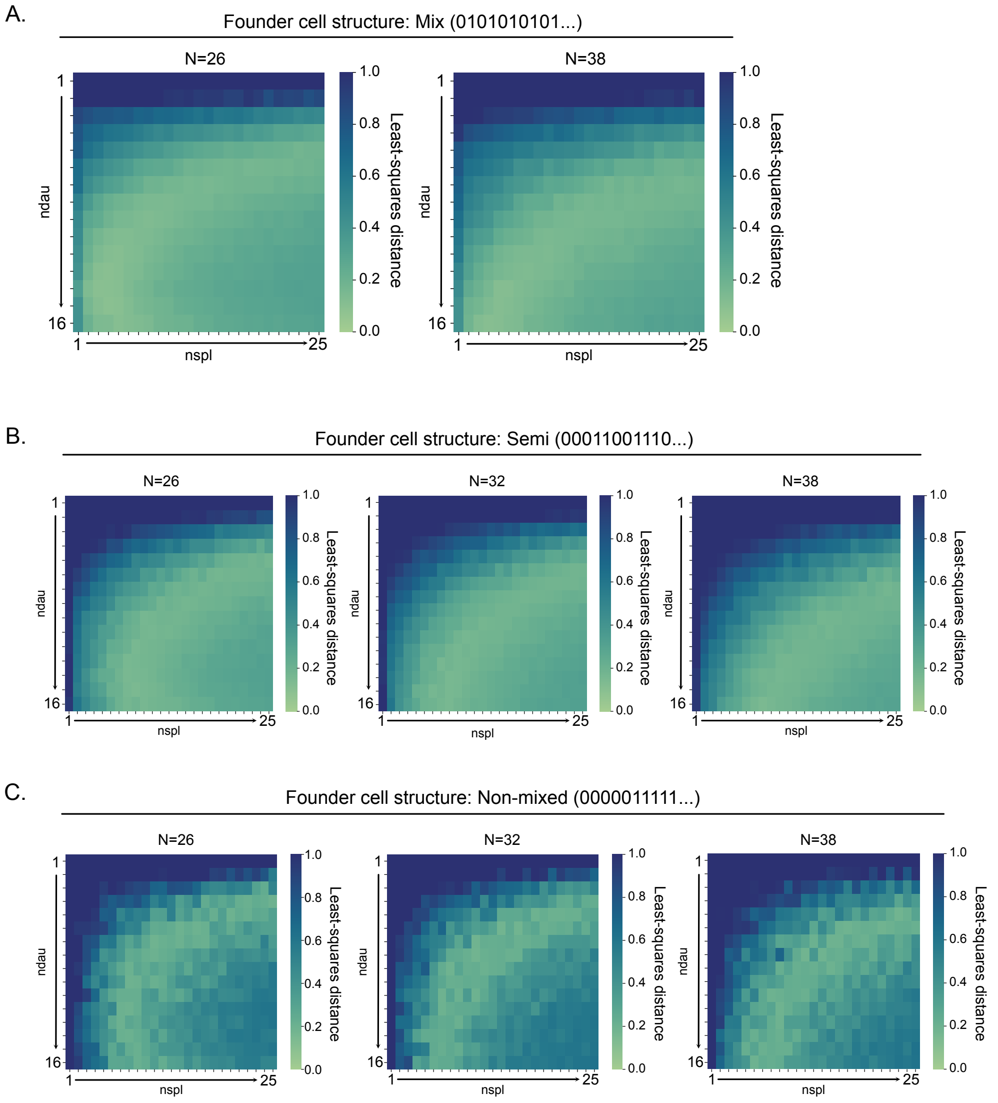

Robustness of the mtDNA segregation model. (A) Simulations for founder cell structure: mixed (0101010101..), and mildly varying the total number of mtDNA copies per cell. Heatmap shows the least-squares distance of simulations with a given pair of *ndau* and *nspl* parameters from the empirical data. Specifically, data represent two outcomes upon modeling

mtDNA segregation where the number of mtDNA molecules per cell prior to division is N=26 or N=38, respectively. *nspl* represents the number of sub-fragments the mother cell will break into , shuffle, and re-fuse prior to division, and *ndau* represents the number of mtDNA copies transferred to the daughter cell upon division. Higher values of the color gradient (blue) indicate larger distances, that is lower likelihood fitting to the data. Each simulation of any combination of parameters has been run 10 times, and the average least-squares distance was taken. **(B)**Simulations for founder cell structure: semi, and mildly varying the total number of mtDNA copies per cell. Heatmap shows the least-squares distance of simulations with a given pair of *ndau* and *nspl* parameters from the empirical data, with a different initial structure for the founder cell (semi: 1110001100011..). Specifically, data represent the outcomes of modeling mtDNA segregation with the number of mtDNA molecules per cell: N=26, N=32 or N=38, respectively. Robustness of the model was tested based on the similarity of the best hits upon varying the total number of copies per cell prior to division, as well as by altering structure of the founder cell. Each simulation of any combination of parameters has been run 10 times, and the average least-squares distance was taken. **(C)** Simulations for founder cell structure: non-mixed, and mildly varying the total number of mtDNA copies per cell. Heatmap shows the least-squares distance of simulations with a given pair of *ndau* and *nspl* parameters from the empirical data, with a different initial structure for the founder cell (non-mixed: 0000011111..). Specifically, data represent the outcomes of modeling mtDNA segregation with the number of mtDNA molecules per cell: N=26, N=32 or N=38, respectively. Robustness of the model was tested based on the similarity of the best hits upon varying the total number of copies per cell prior to division, as well as by altering the structure of the founder cell. Each simulation of any combination of parameters has been run 10 times, and the average least-squares distance was taken.

Appendix Figure S8

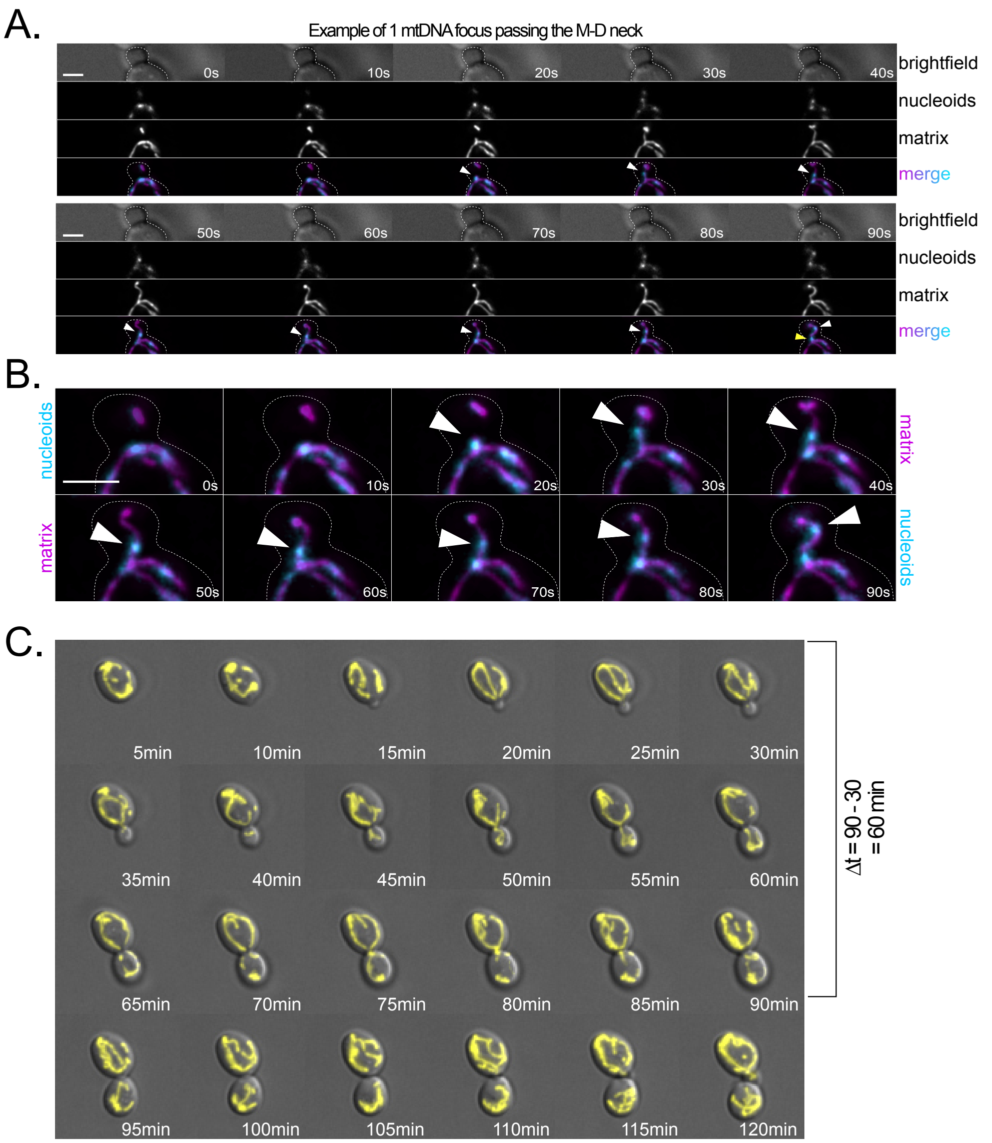

Inherited mtDNA foci and mitochondrial network connectivity of mother-bud pairs. (A) Representative images of one mother-bud pair in early S phase. Using the mtLacO-LacI system, each nucleoid is visible as a fluorescent foci, migrating across the bud-neck in the 5-minute window.

Nucleoids (cyan) are assumed to contain only one mtDNA copy. Mitochondrial network is shown in magenta. Log-phase cells from different cell cycle stages were examined (n=93 mother-bud pairs). Fluorescence images are maximum intensity projections of z-stacks, after deconvolution. Scale bars: 5 μm. **(B)** Enlarged images of the merge of S7A. Nucleoids represented in cyan, mitochondrial network in magenta. White arrows indicate one focus crossing the mother-bud neck. Scale bars: 5 μm. **(C)** Example timelapse images of a mother-bud pair with total mitochondrial network connectivity duration $\Delta t = 60$ minutes. Cells expressing the construct su9-matrix-mKate2 were used to visualize the mitochondrial network (yellow). Only cells in late G1 or early S phase were considered for the calculation, that is no visible bud by the start of imaging (n=33 cells).

Appendix Figure S9

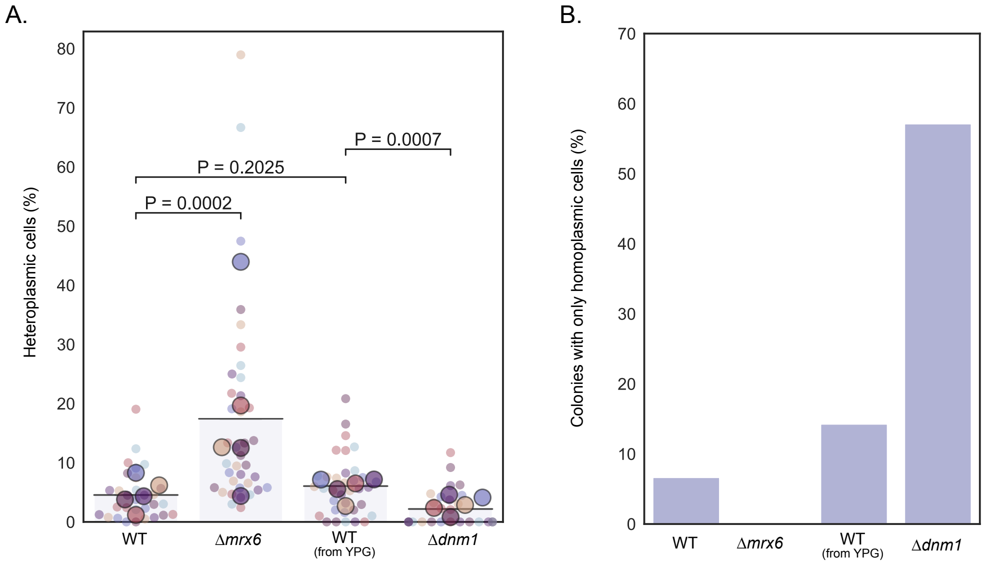

18-hr heteroplasmy assessment in Δ*mrx6* and Δ*dnm1* deletion strains. **(A)** Heteroplasmy levels in matings of Δ*mrx6* and Δ*dnm1* deletion strains. Cells with increased copy number (Δ*mrx6*) or fission-deficient (Δ*dnm1*) cells were generated in strains containing mtDNA$^{\text{Atp6-NG}}$ or mtDNA$^{\text{Atp6-mKate2}}$. After zygote formation and microdissection, diploid colonies were grown for 18 hrs on a YPD plate. Cells from each colony were imaged to assess the fraction of heteroplasmic cells (see also: Fig. S8). Of note, Δ*dnm1* cells were cultivated in YPG media, to prevent any mtDNA loss, before mating and dissection on YPD plates. For each genotype shown, the small dots represent the mean percentage of heteroplasmic cells in an individual colony (N>50 cells/colony). Each bigger dot depicts the mean of each biological replicate (N=5). The line represents the mean of all replicates for the corresponding mating. Statistical significance was determined by paired t-test. **(B)** Barplot of all populations derived from individual heteroplasmic zygotes, where no heteroplasmic cells were detected across all replicates, after 18 hrs. Mating of Δ*dnm1* cells resulted in a high percentage of populations, where only homoplasmic cells were detected. In contrast, for matings of

Δ*mrx6* cells, heteroplasmic cells were detected in all populations.

Appendix Table S1: Yeast Strains

Name	Genotype	Origin
yCO844	ade2-1 his3-11,15 trp1-1 leu2-3,112 ura3-1 CAN1 arg8::HIS3 MAT a (MR6)	Rak et al., 2006
yCO845	ade2-1 his3-11,15 trp1-1 leu2-3,112 ura3-1 CAN1 arg8::HIS3- Δ*atp6* MAT a (MR10)	Rak et al., 2006
yCO084	ade2-1 his3-11,15 trp1-1 leu2-3,112 ura3-1 CAN1 arg8::HIS3 ATP6-NeonGreen MAT alpha	Jakubke et al., 2021
yRR043	ade2-1 his3-11,15 trp1-1 leu2-3,112 ura3-1 CAN1 arg8::HIS3 ATP6-mKate2 MAT a	Jakubke et al., 2021
yFT019	leu2-3,112 can1-100 ura3-1 his3-11,15 Leu2-Pcup1-3xNG-LacI-PGK1-Su9-mKate2-Leu2 MAT a/alpha	this study
yRR195	ade2-1 his3-11,15 trp1-1 leu2-3,112 ura3-1 CAN1 arg8::HIS3 ATP6-NeonGreen Δ*pdr5*::Hyg MAT alpha	this study
yRR194	ade2-1 his3-11,15 trp1-1 leu2-3,112 ura3-1 CAN1 arg8::HIS3 ATP6-mKate2 Δ*pdr5*::Hyg MAT a	this study
yRR202	ade2-1 his3-11,15 trp1-1 leu2-3,112 ura3-1 CAN1 arg8::HIS3 ATP6-NeonGreen Δ*abf2*::Hyg MAT alpha	this study
yRR203	ade2-1 his3-11,15 trp1-1 leu2-3,112 ura3-1 CAN1 arg8::HIS3 ATP6-mKate2 Δ*abf2*::Hyg MAT a	this study
yRR187	ade2-1 his3-11,15 trp1-1 leu2-3,112 ura3-1 CAN1 arg8::HIS3 ATP6-NeonGreen Δ*dnm1*::Hyg MAT alpha	this study
yRR186	ade2-1 his3-11,15 trp1-1 leu2-3,112 ura3-1 CAN1 arg8::HIS3 ATP6-mKate2 Δ*dnm1*::Hyg MAT a	this study
yRR20	ade2-1 his3-11,15 trp1-1 leu2-3,112 ura3-1 CAN1 arg8::HIS3 ATP6-NeonGreen Δ*mrx6*::Nat MAT alpha	this study
yRR206	ade2-1 his3-11,15 trp1-1 leu2-3,112 ura3-1 CAN1 arg8::HIS3 ATP6-mKate2 Δ*mrx6*::Nat MAT a	this study
yCO354	ade2-1 his3-11,15 trp1-1 leu2-3,112 ura3-1 CAN1 arg8::HIS3 cob::ARG8M intronless mtDNA MAT alpha	Gruschke et al., 2011
yCO391	ade2-1 his3-11,15 trp1-1 leu2-3,112 ura3-1 CAN1 arg8::HIS3 intronless mtDNA MAT a	Gruschke et al., 2011

Appendix Table S2: Oligonucleotides

Name	Sequence	Purpose
CO2836	TTAAGTTTTCGTATCCGCTCGTTCGAAAGACTTT AGACAAAAATGCGTACGCTGCAGGTCGAC	PDR5 fwd (S1)
CO2837	CATCTTGGTAAGTTTCTTTTCTTAACCAAATTCA AAATTCTATTAATCGATGAATTCGAGCTCG	PDR5 rev (S2)
CO1158	GTAAACAGATTAACAAAGAAGCCAATCAATTACA ACAACAAATAACGTACGCTGCAGGTCGAC	ABF2 fwd (S1)
CO1159	ACGGAAAGAATAAAGGCATAAAAAACATTGTGAG AGTACCGCGGTATCGATGAATTCGAGCTCG	ABF2 rev (S2)
CO356	CATTAAGTAGCTACCAGCGAATCTAAATACGACG GATAAAGAATGCGTACGCTGCAGGTCGAC	DNM1 fwd (S1)
CO357	ACGCAATGTTGAAGTAAGATCAAAAATGAGATGA ATTATGCAATTAATCGATGAATTCGAGCTCG	DNM1 rev (S2)
CO870	TATGTCACATAATAGAGCAGTGGATAAGTACTCA ATGCAAAATCAATCGATGAATTCGAGCTCG	MRX6 fwd (S1)
CO914	AGATCATTGCAGAAGTAGTGAGATTTAGTCGTA CGTTACGTCATGCGTACGCTGCAGGTCGAC	MRX6 rev (S2)

Appendix Table S3: Plasmids

Name	Insert	Selection marker	Origin
pCO059	pFA6a-NatNT2	Nourseothricin	Janke et al., 2004
pCO021	pfa6a-kanMX6	G418	Janke et al., 2004
pCO074	pfa6a-hphNT1	Hygromycin	Janke et al., 2004
pCO494	pKT127-NeonGreen (Janke primers)	G418	Osman et al., 2015
pCO533	pKT127-mKate2 (Janke primers)	G418	Jakubke et al., 2021
