# Supplementary figures and images for: Real-time assessment of mitochondrial DNA heteroplasmy dynamics at the single-cell level
Source: EMBO J. 2024 Aug 5;43(22):4. doi: 10.1038/s44318-024-00183-5 (PMC11574196; doi:10.1038/s44318-024-00183-5)

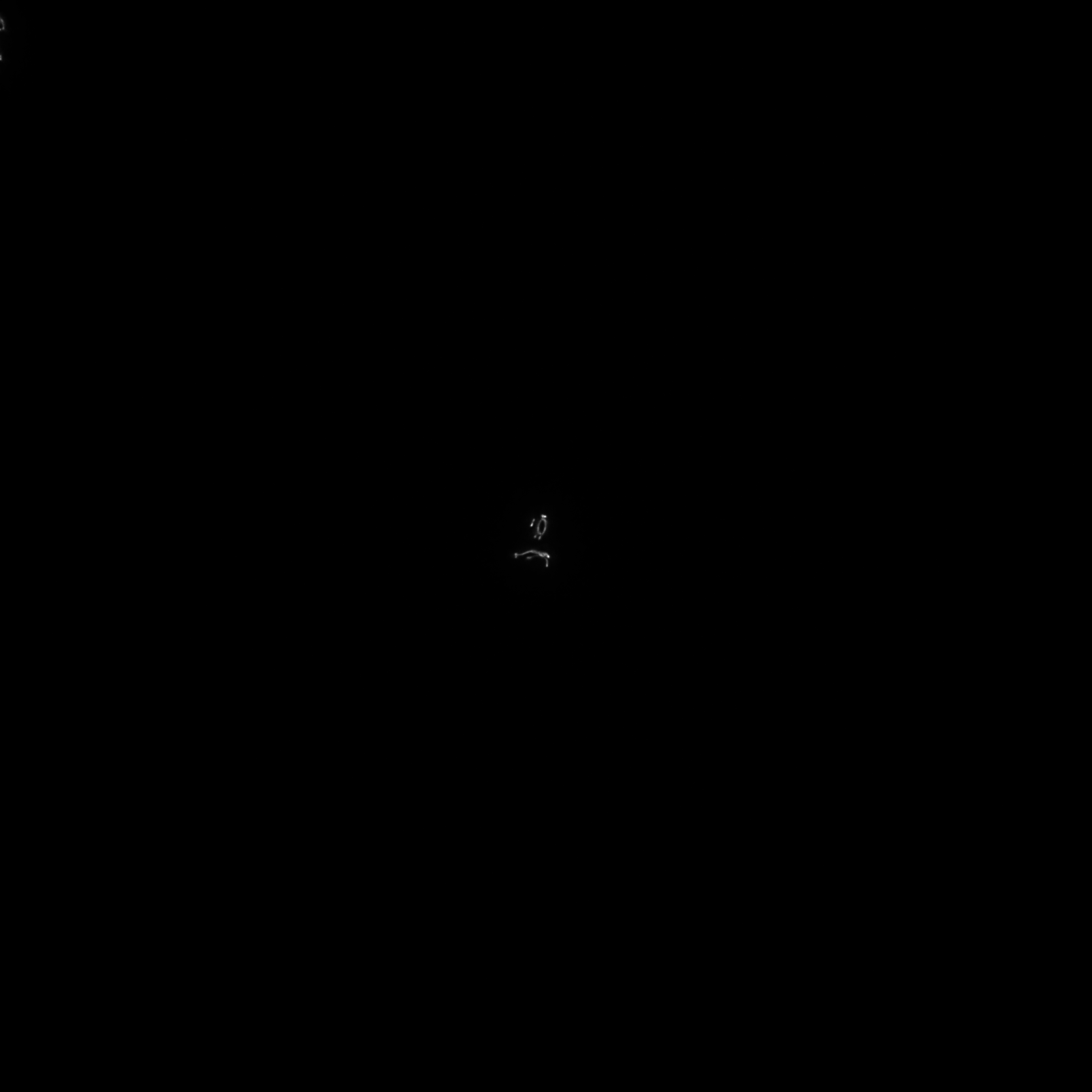

Supplement: Supplementary file 15 — Source data Fig. 2 [file 44318_2024_183_MOESM15_ESM.zip › Figure 2/2A/NG/MAX_131222_Atp6_ng_RR_series003_NG_fr0.tif]

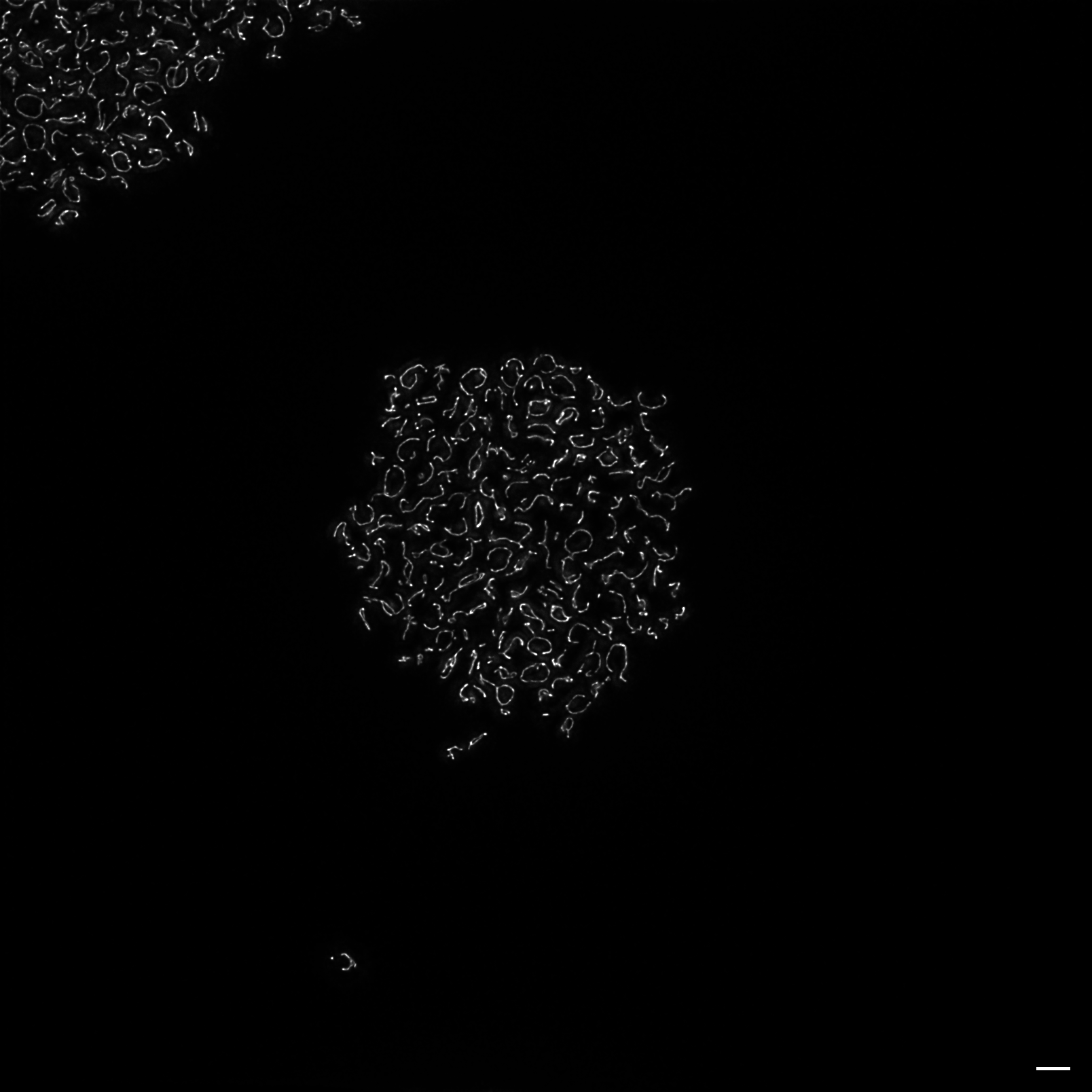

Supplement: Supplementary file 15 — Source data Fig. 2 [file 44318_2024_183_MOESM15_ESM.zip › Figure 2/2A/NG/MAX_131222_Atp6_ng_RR_series003_NG_fr32_5umscalebar.tif]

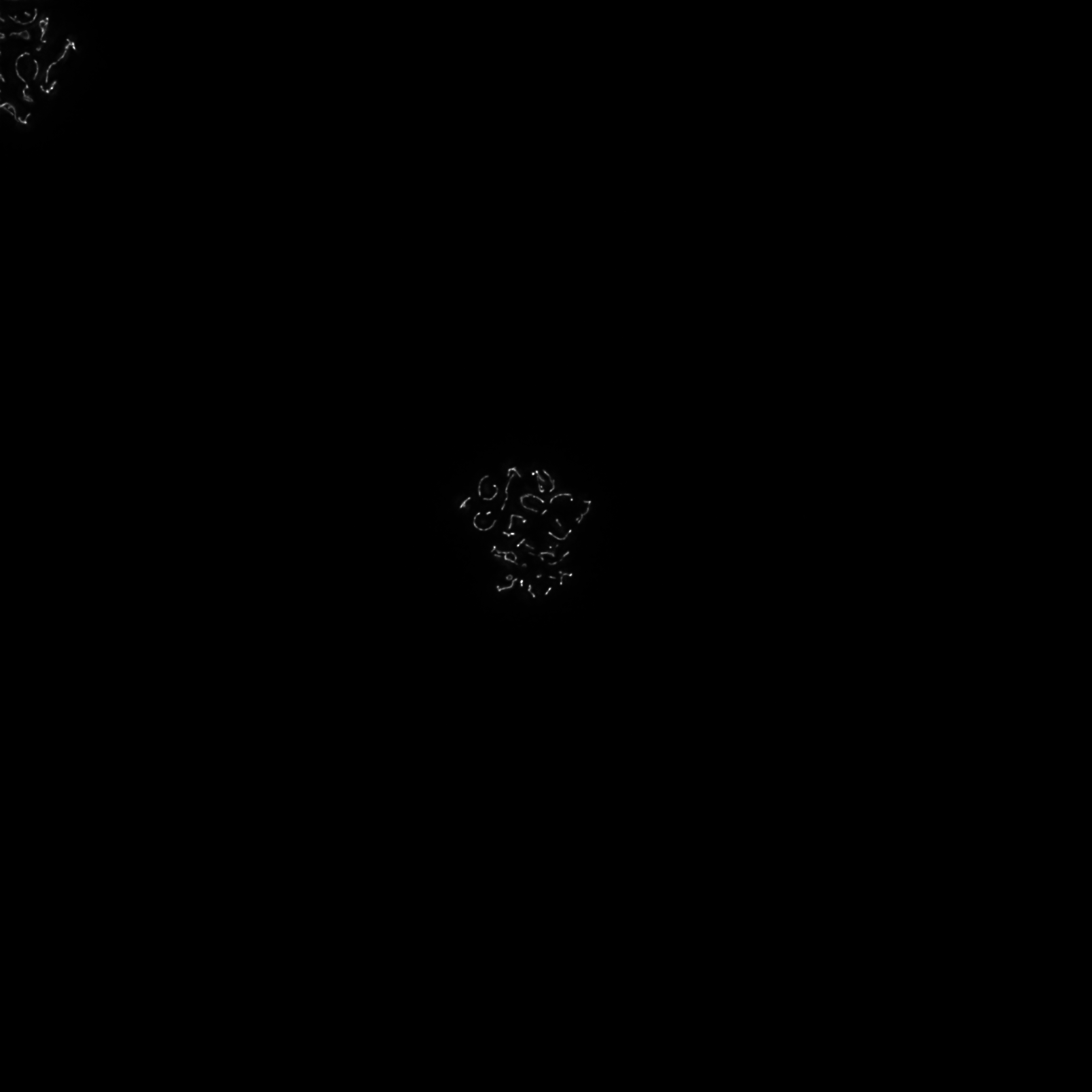

Supplement: Supplementary file 15 — Source data Fig. 2 [file 44318_2024_183_MOESM15_ESM.zip › Figure 2/2A/NG/MAX_131222_Atp6_ng_RR_series003_NG_fr16.tif]

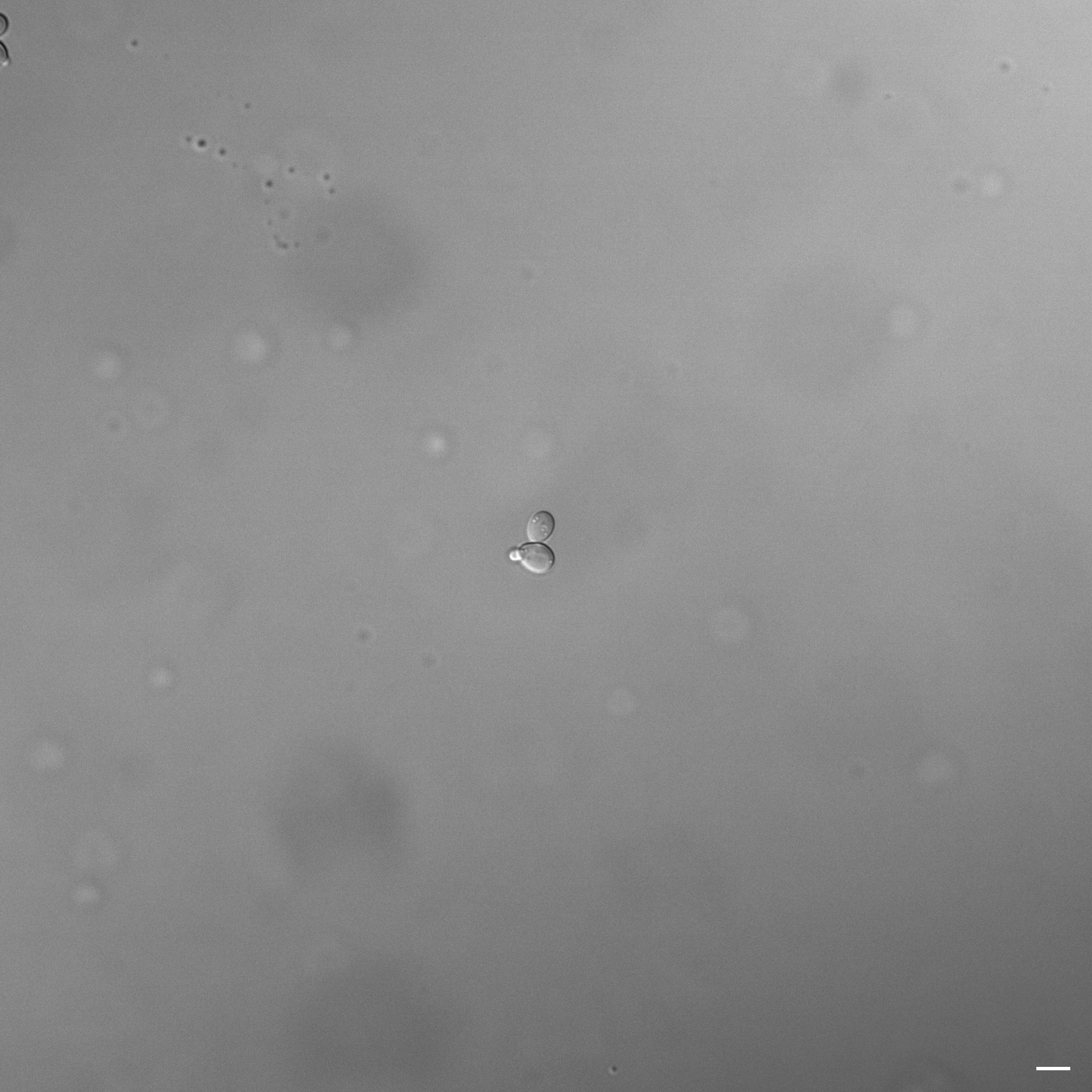

Supplement: Supplementary file 15 — Source data Fig. 2 [file 44318_2024_183_MOESM15_ESM.zip › Figure 2/2A/BF/131222_Atp6_ng_RR_series003_BF_z11_fr0_5umscalebar.tif]

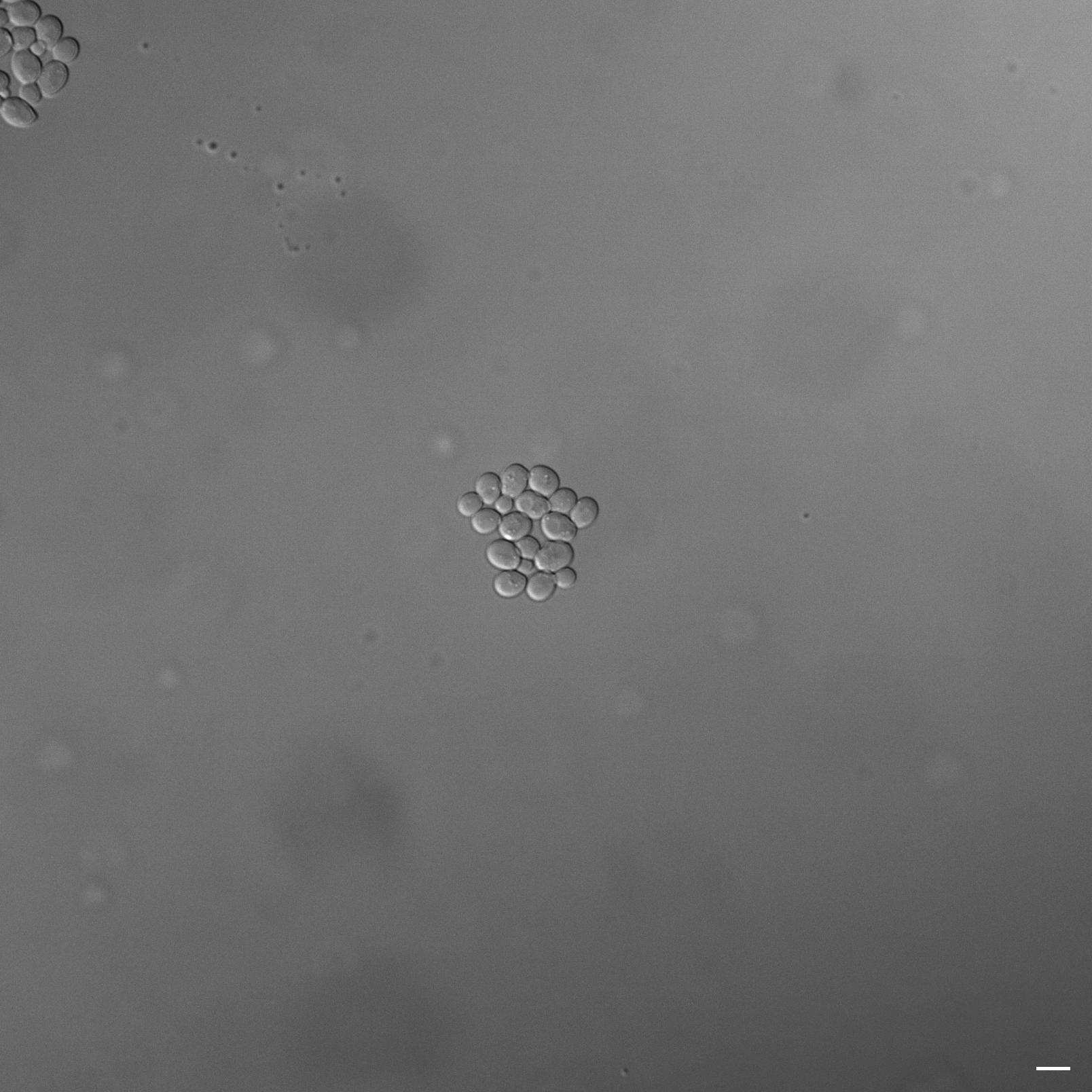

Supplement: Supplementary file 15 — Source data Fig. 2 [file 44318_2024_183_MOESM15_ESM.zip › Figure 2/2A/BF/131222_Atp6_ng_RR_series003_BF_z11_fr16_5umscalebar.tif]

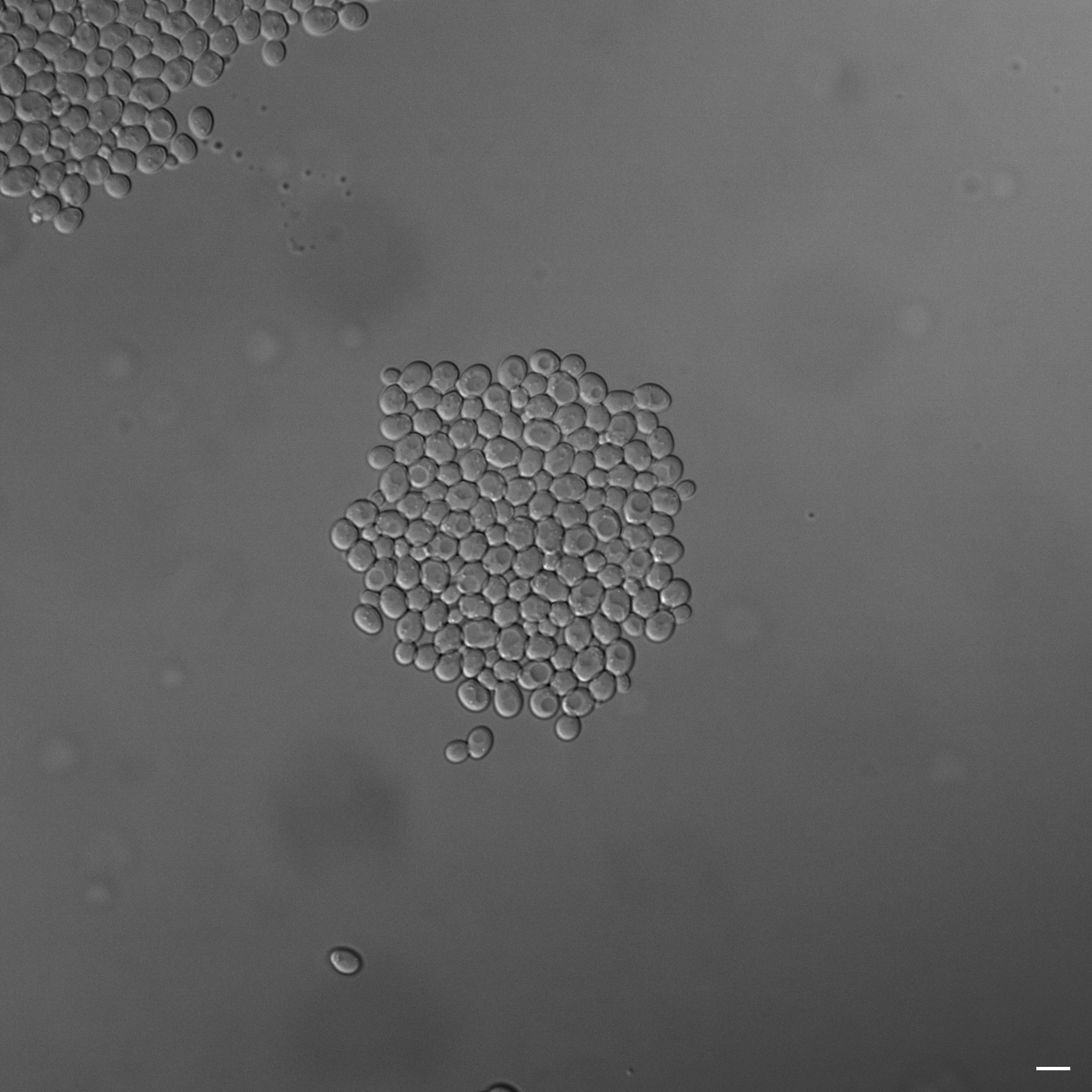

Supplement: Supplementary file 15 — Source data Fig. 2 [file 44318_2024_183_MOESM15_ESM.zip › Figure 2/2A/BF/131222_Atp6_ng_RR_series003_BF_z11_fr32_5umscalebar.tif]

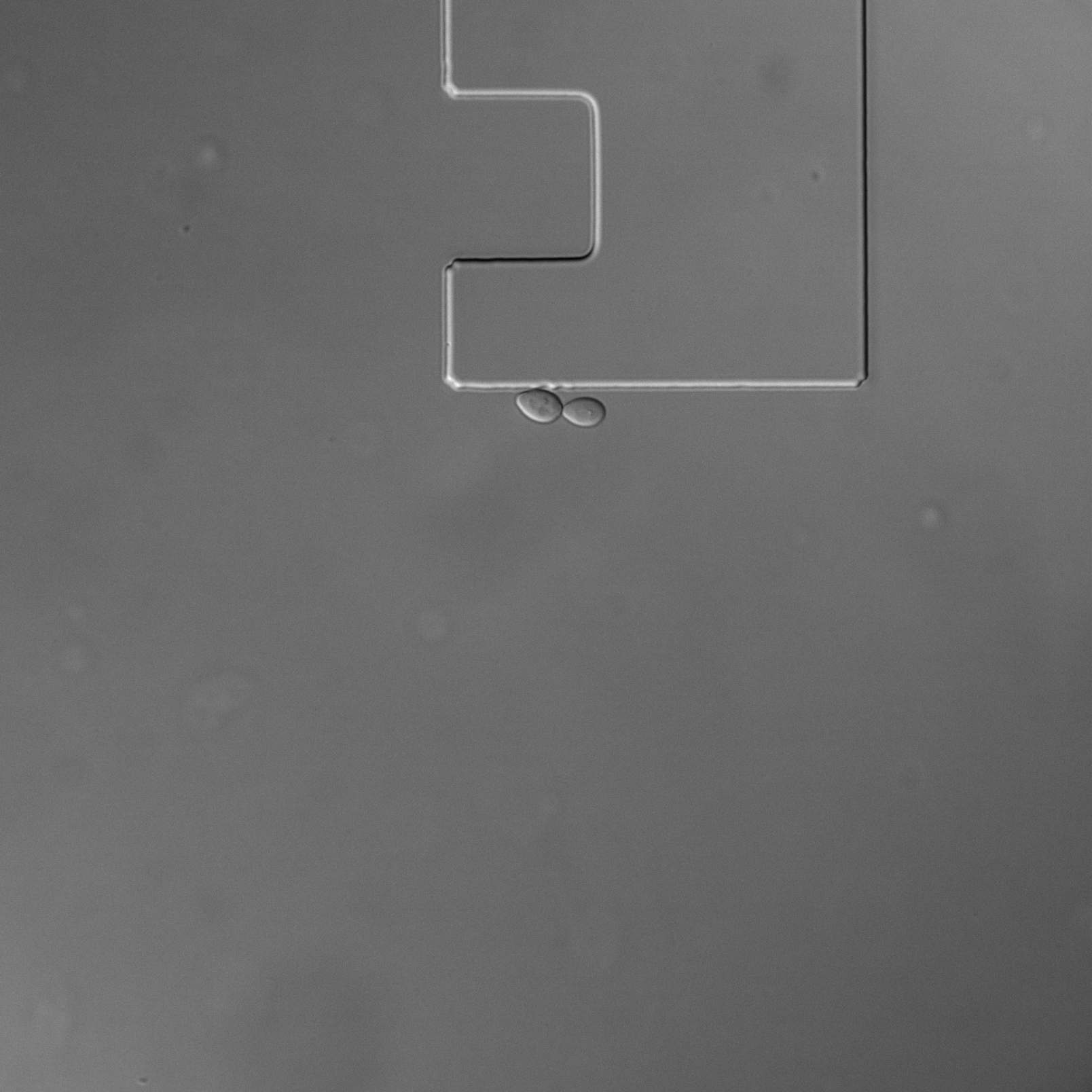

Supplement: Supplementary file 15 — Source data Fig. 2 [file 44318_2024_183_MOESM15_ESM.zip › Figure 2/2B/BF/MAX_200922_Atp6_mKate2_Haploid_series008_BF_f0.tif]

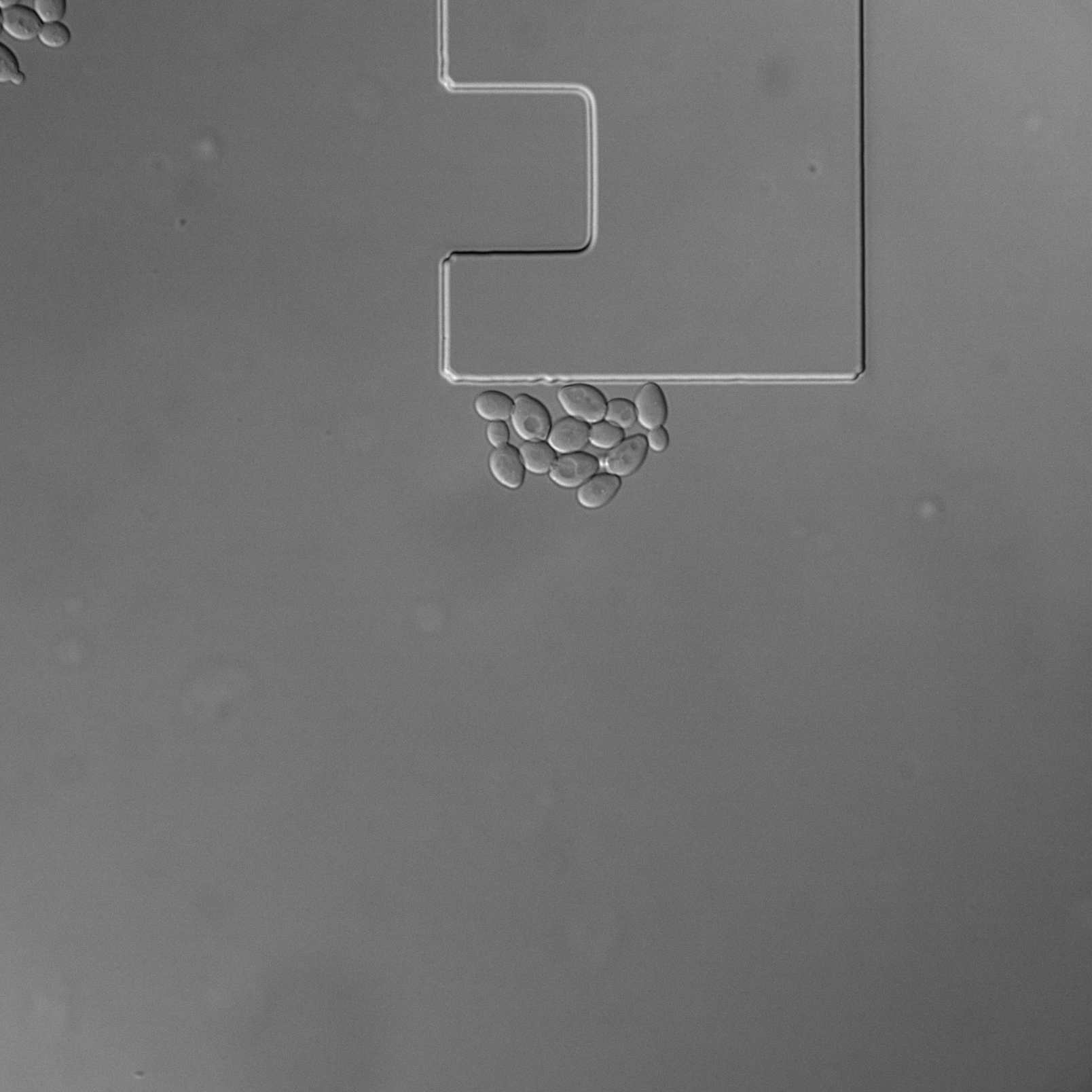

Supplement: Supplementary file 15 — Source data Fig. 2 [file 44318_2024_183_MOESM15_ESM.zip › Figure 2/2B/BF/MAX_200922_Atp6_mKate2_Haploid_series008_BF_f16.tif]

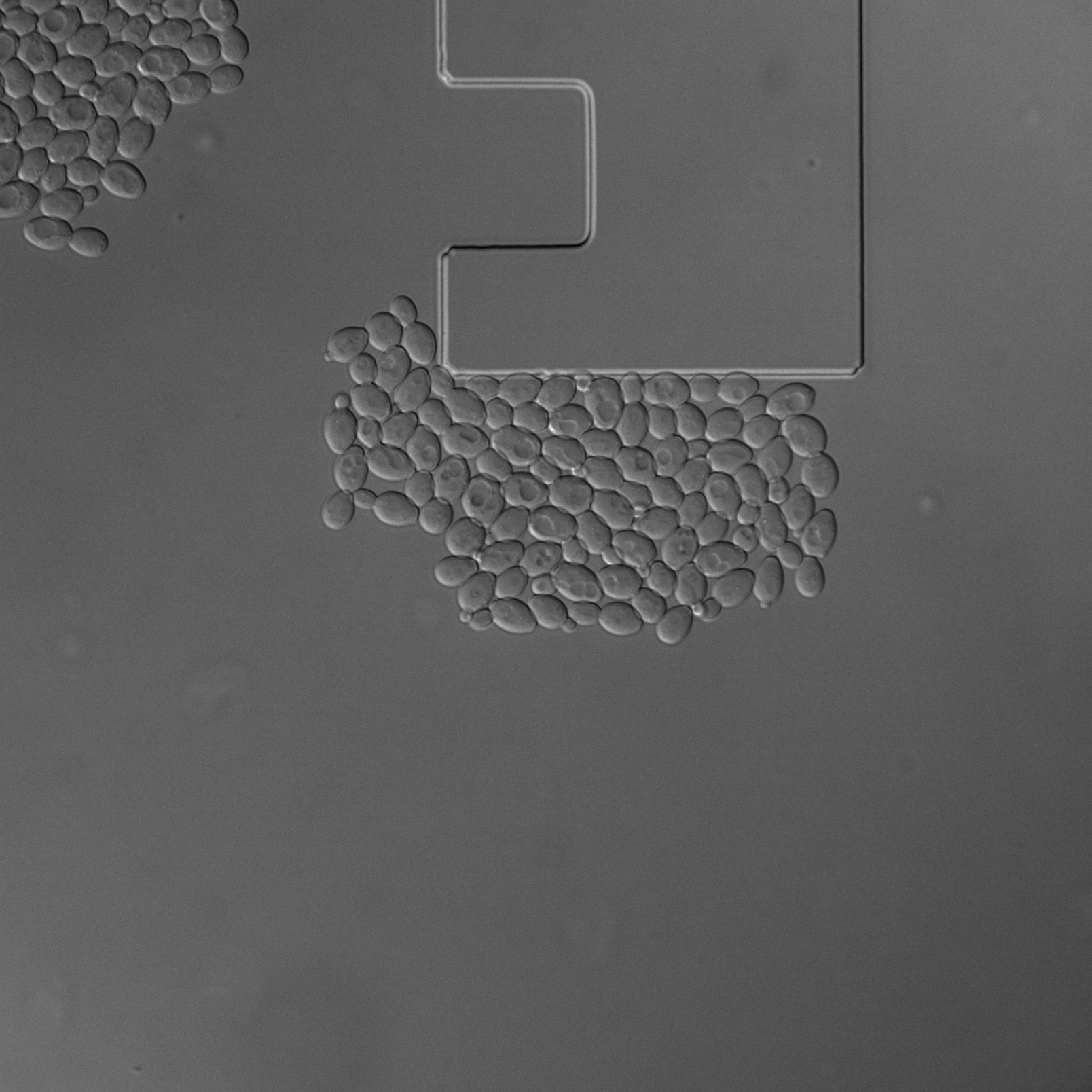

Supplement: Supplementary file 15 — Source data Fig. 2 [file 44318_2024_183_MOESM15_ESM.zip › Figure 2/2B/BF/MAX_200922_Atp6_mKate2_Haploid_series008_BF_f32.tif]

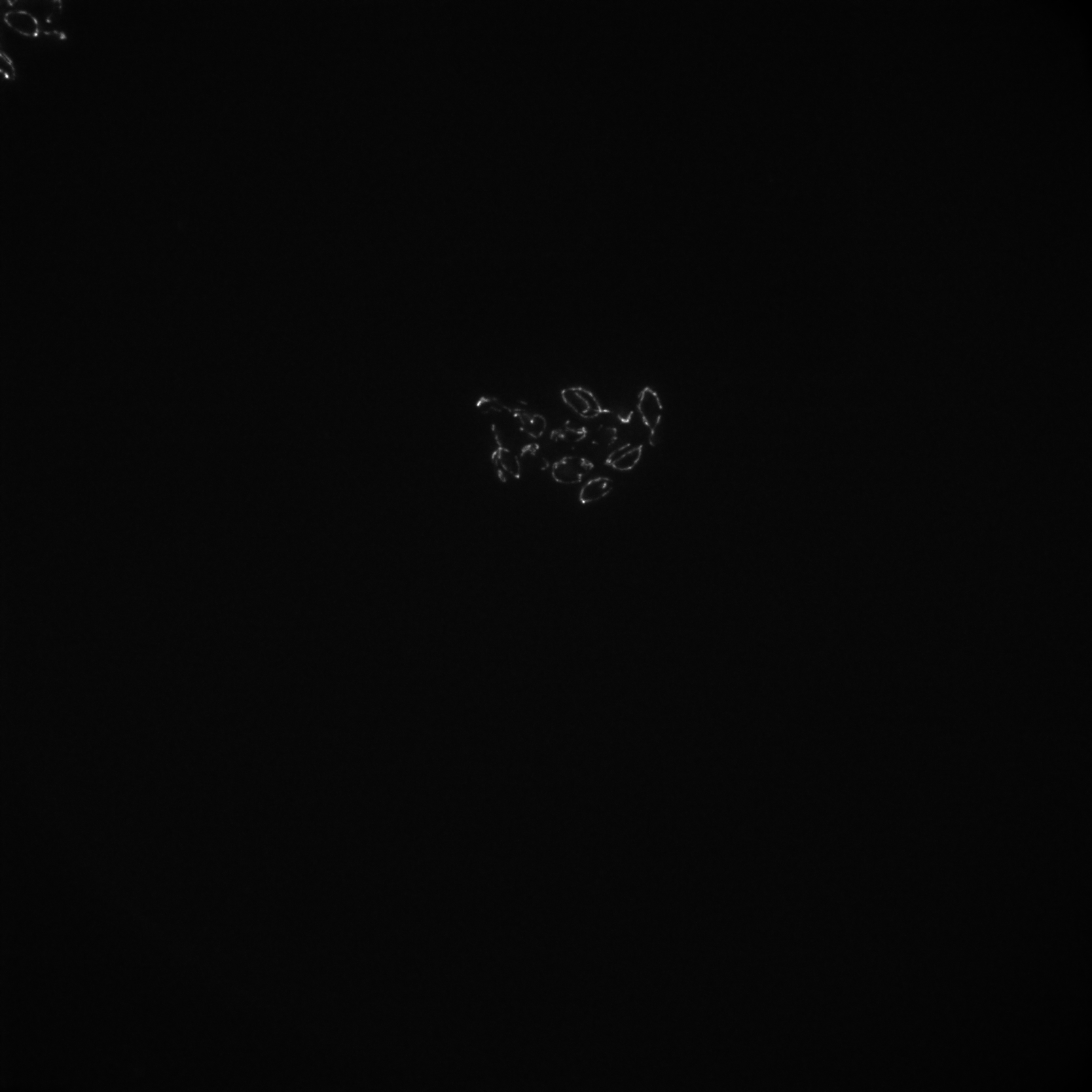

Supplement: Supplementary file 15 — Source data Fig. 2 [file 44318_2024_183_MOESM15_ESM.zip › Figure 2/2B/mKate2/MAX_200922_Atp6_mKate2_Haploid_series008_mKate_f16.tif]

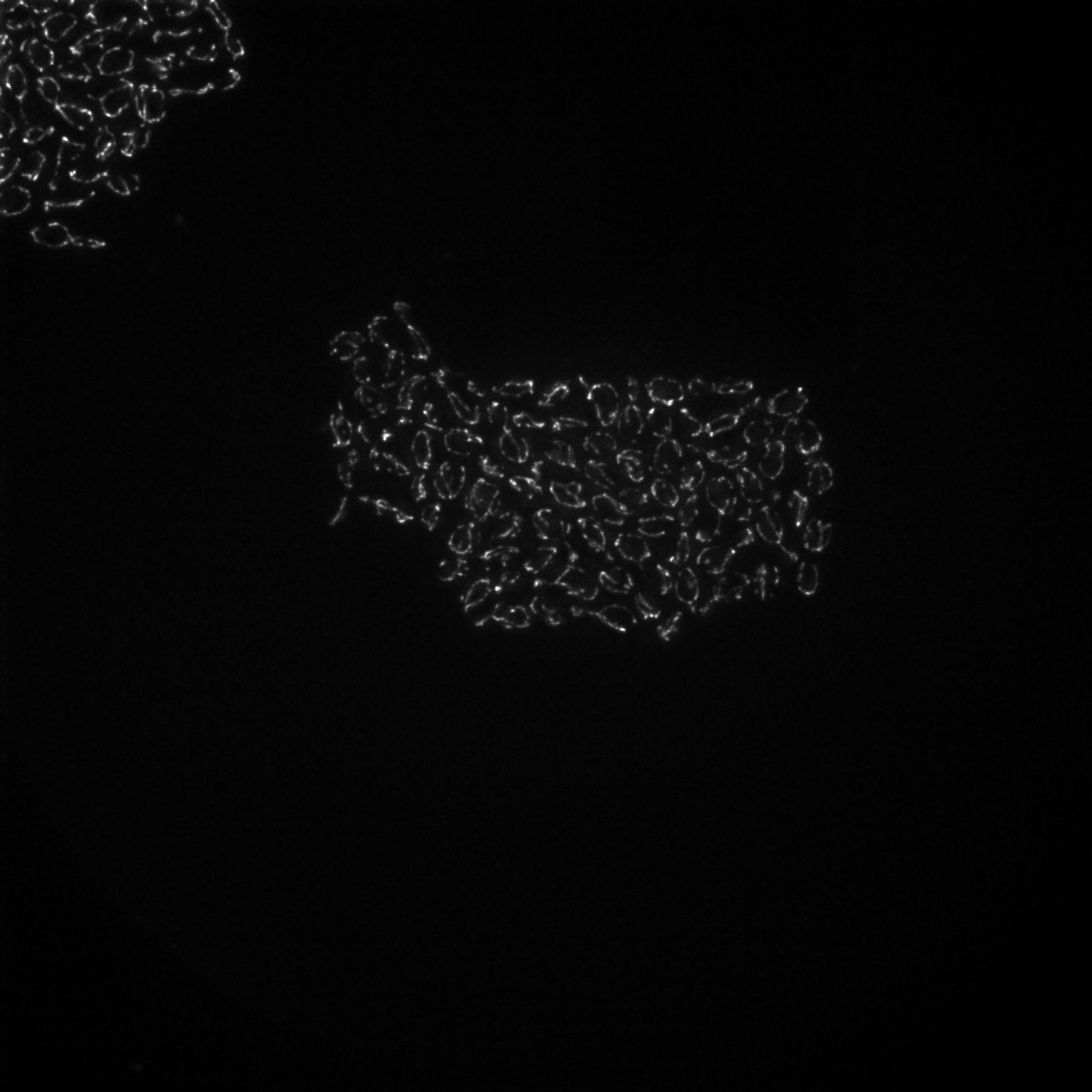

Supplement: Supplementary file 15 — Source data Fig. 2 [file 44318_2024_183_MOESM15_ESM.zip › Figure 2/2B/mKate2/MAX_200922_Atp6_mKate2_Haploid_series008_mKate_f32.tif]

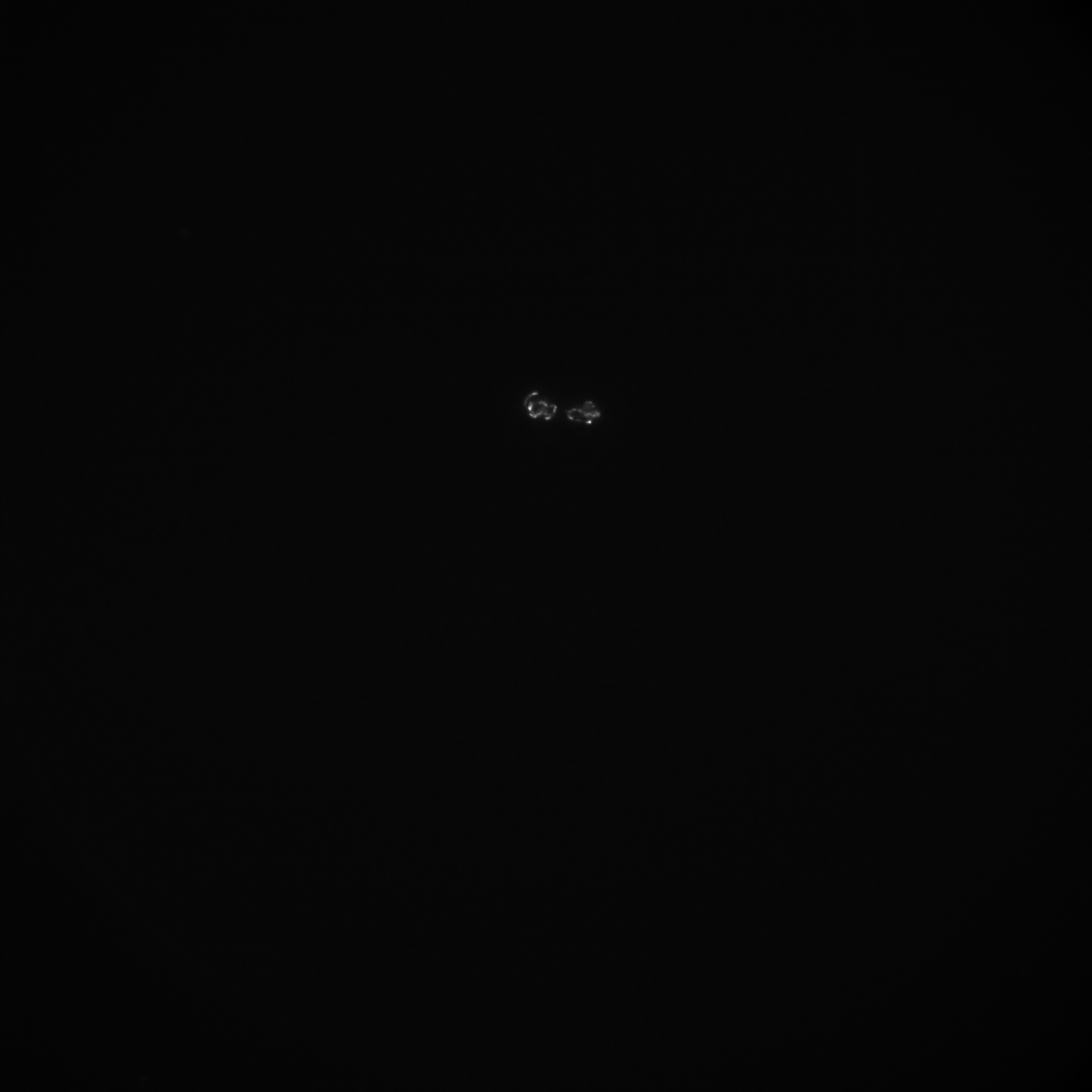

Supplement: Supplementary file 15 — Source data Fig. 2 [file 44318_2024_183_MOESM15_ESM.zip › Figure 2/2B/mKate2/MAX_200922_Atp6_mKate2_Haploid_series008_mKate_fr0.tif]

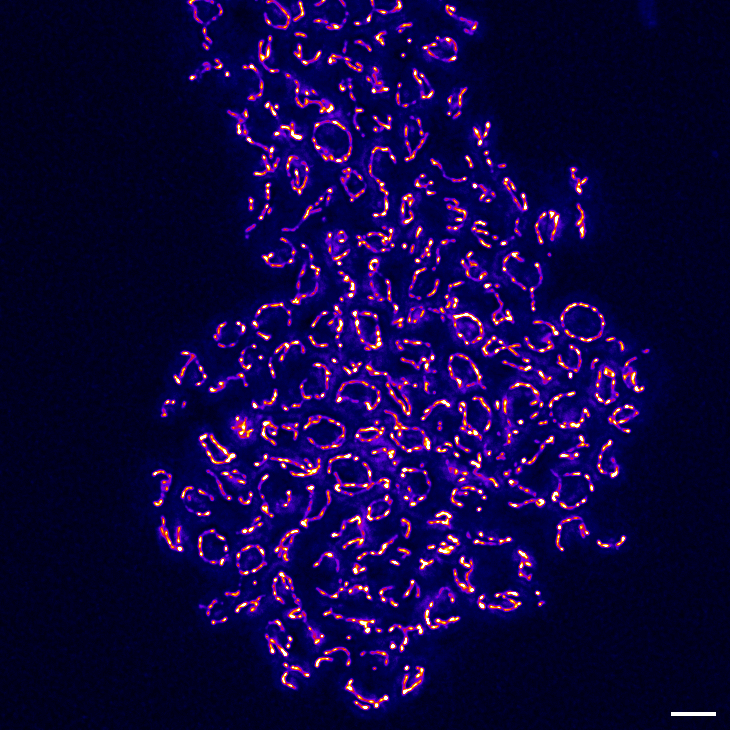

Supplement: Supplementary file 15 — Source data Fig. 2 [file 44318_2024_183_MOESM15_ESM.zip › Figure 2/2C/wt_ypg_mKate2/101123_atp6_mkate_ypg_series05_T40_mKate_scalebar5um_.tif]

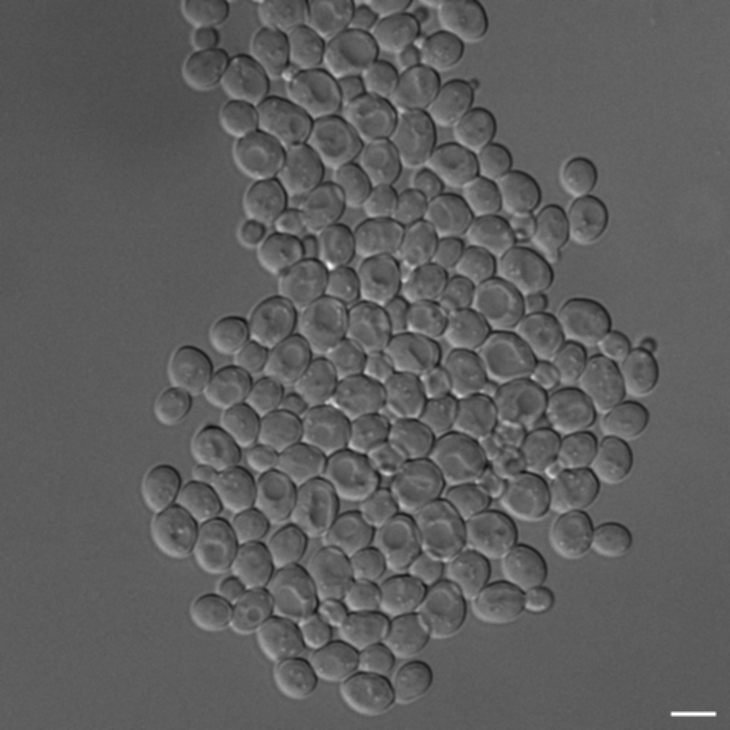

Supplement: Supplementary file 15 — Source data Fig. 2 [file 44318_2024_183_MOESM15_ESM.zip › Figure 2/2C/wt_ypg_mKate2/101123_atp6_mkate_ypg_series05_T40_BF_scalebar5um_.tif]

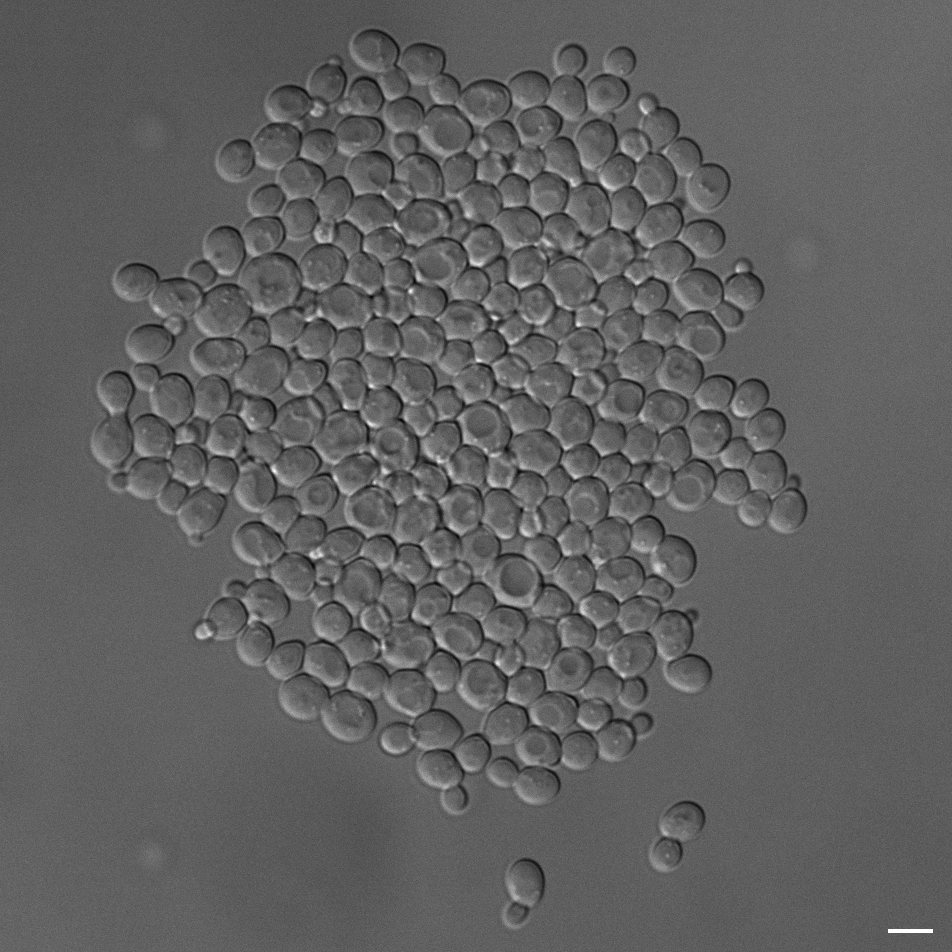

Supplement: Supplementary file 15 — Source data Fig. 2 [file 44318_2024_183_MOESM15_ESM.zip › Figure 2/2C/wt_ypg_NG/ypg_atp6_ng_12hrs_091124_series05_T40_BF_scalebar5um_.tif]

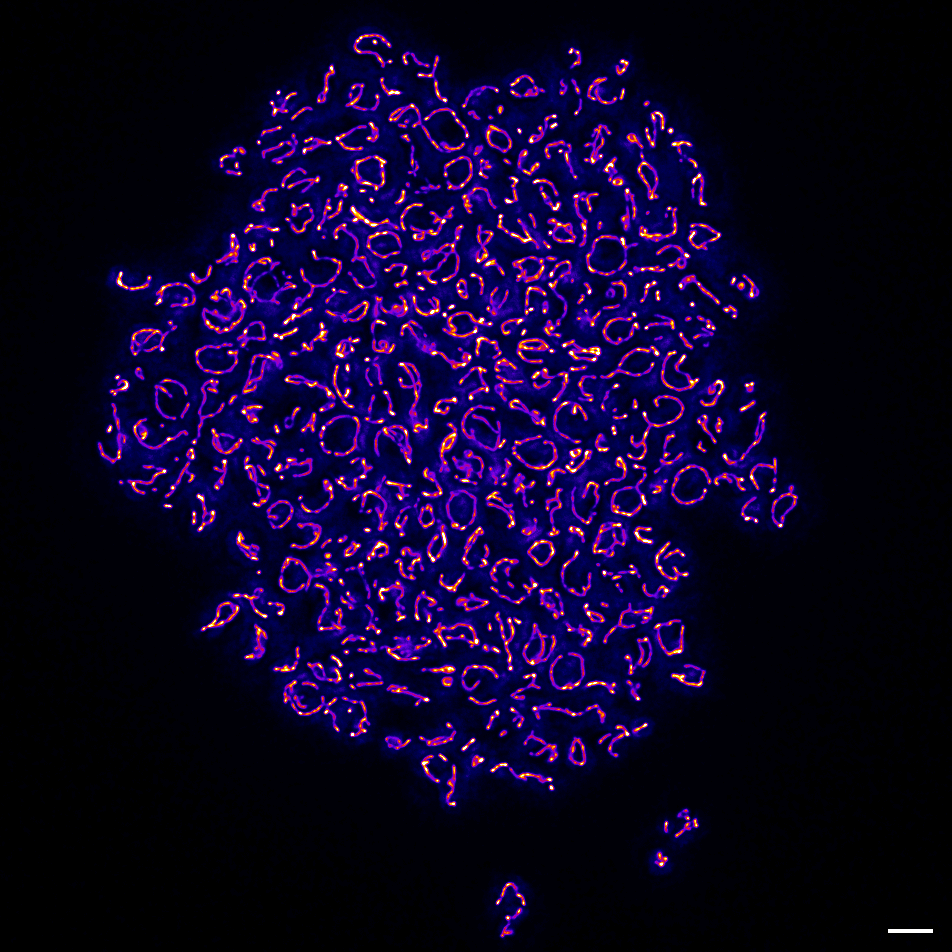

Supplement: Supplementary file 15 — Source data Fig. 2 [file 44318_2024_183_MOESM15_ESM.zip › Figure 2/2C/wt_ypg_NG/ypg_atp6_ng_12hrs_091124_series05_T40_NG_scalebar5um_.tif]

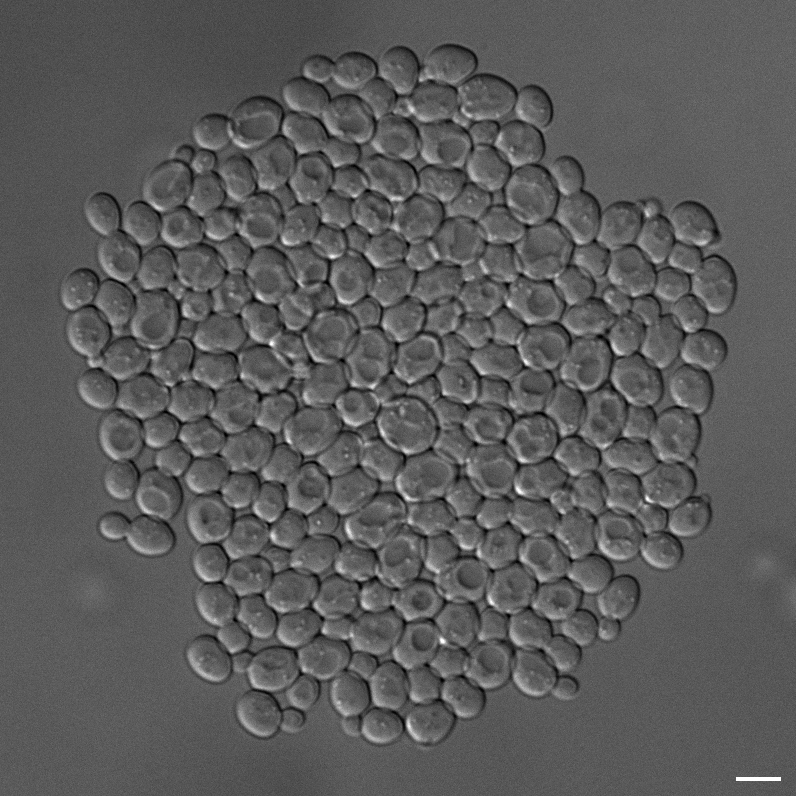

Supplement: Supplementary file 15 — Source data Fig. 2 [file 44318_2024_183_MOESM15_ESM.zip › Figure 2/2C/Γêåabf2_mKate2/MAX_deltaabf2_atp6mk_14hrs_130923_series07_T40_BF_scalebar5um_.tif]

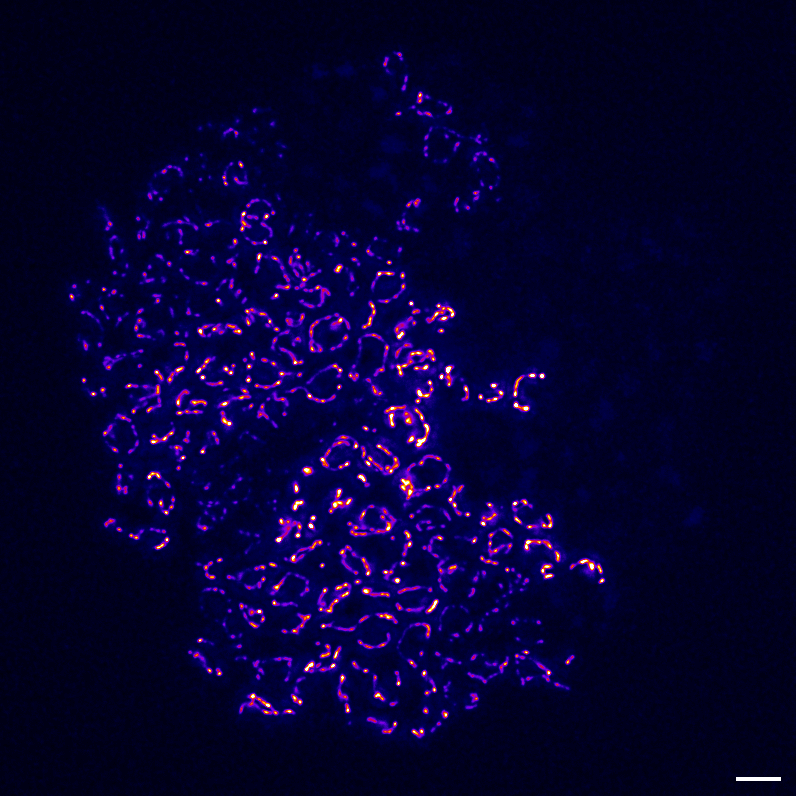

Supplement: Supplementary file 15 — Source data Fig. 2 [file 44318_2024_183_MOESM15_ESM.zip › Figure 2/2C/Γêåabf2_mKate2/MAX_deltaabf2_atp6mk_14hrs_130923_series07_T40_mKate_scalebar5um_.tif]

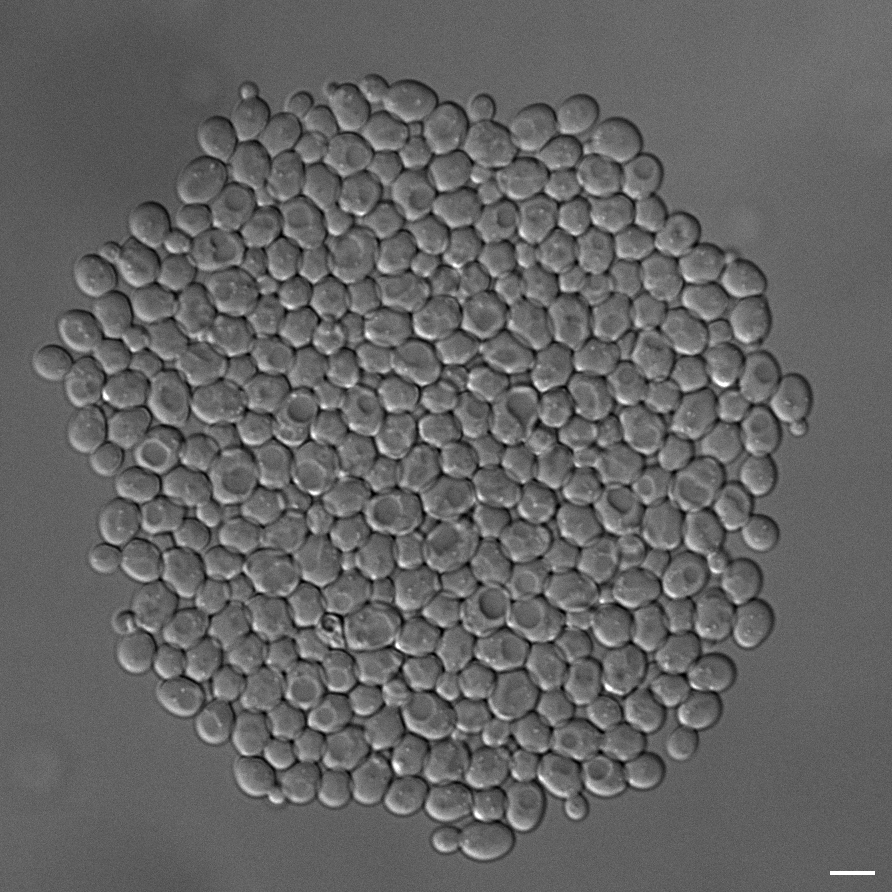

Supplement: Supplementary file 15 — Source data Fig. 2 [file 44318_2024_183_MOESM15_ESM.zip › Figure 2/2C/Γêåabf2_NG/deltaabf2_atp6ng_14hrs_140924_series4_BF_t40_5umscalebar_.tif]

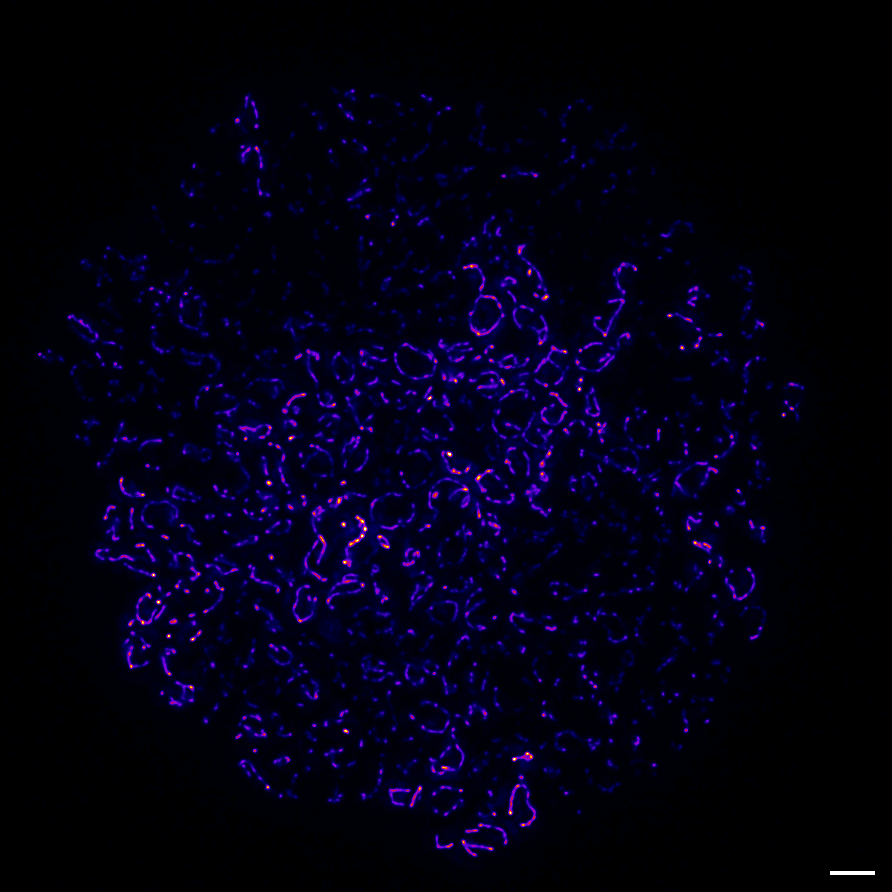

Supplement: Supplementary file 15 — Source data Fig. 2 [file 44318_2024_183_MOESM15_ESM.zip › Figure 2/2C/Γêåabf2_NG/deltaabf2_atp6ng_14hrs_140924_series4_NG_t40_5umscalebar_.tif]

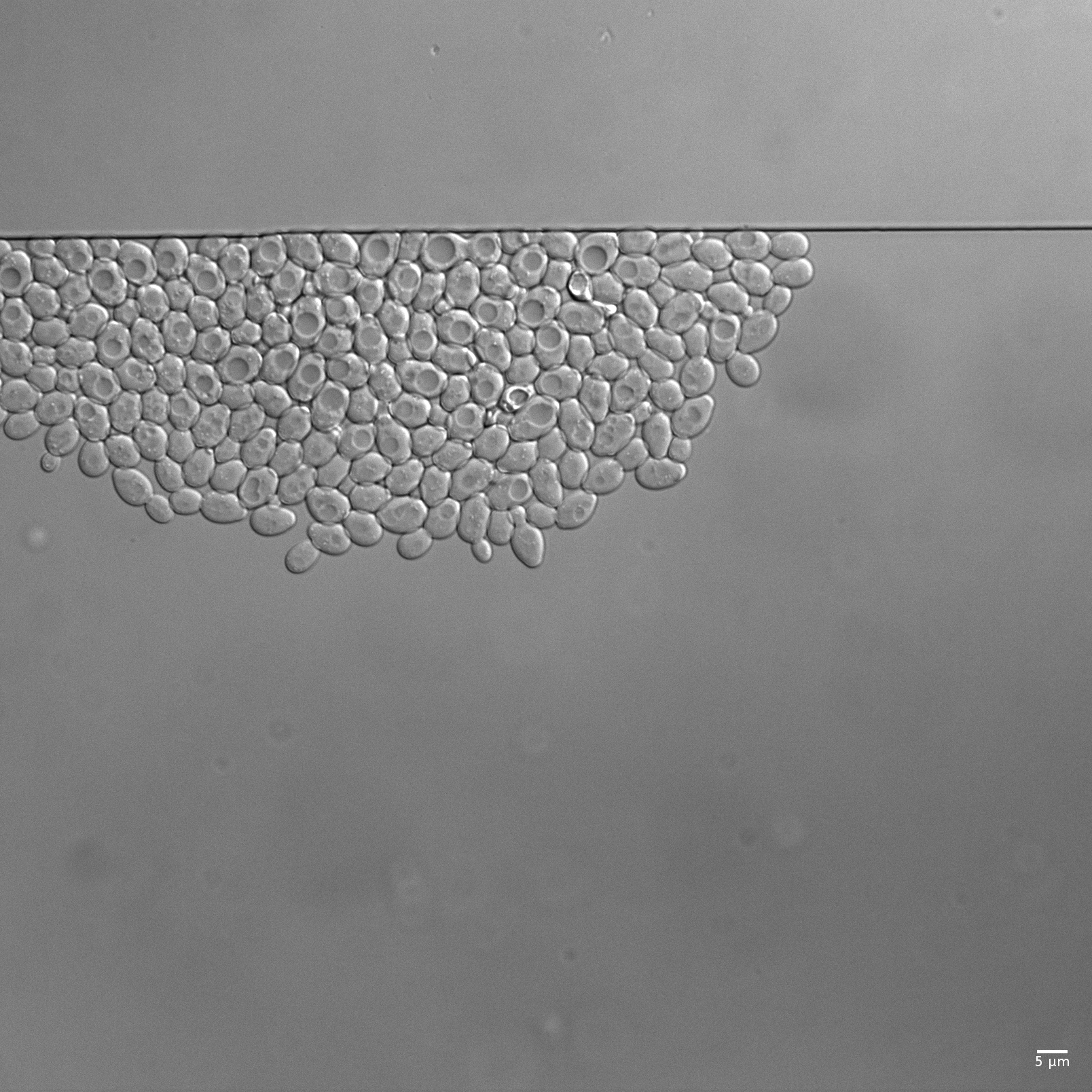

Supplement: Supplementary file 16 — Source data Fig. 3 [file 44318_2024_183_MOESM16_ESM.zip › Figure 3/3A/BF/060223_wt_ng_mk.cl1_series005_T032.ome_cmle_BF_fr32_use_scalebar.tif]

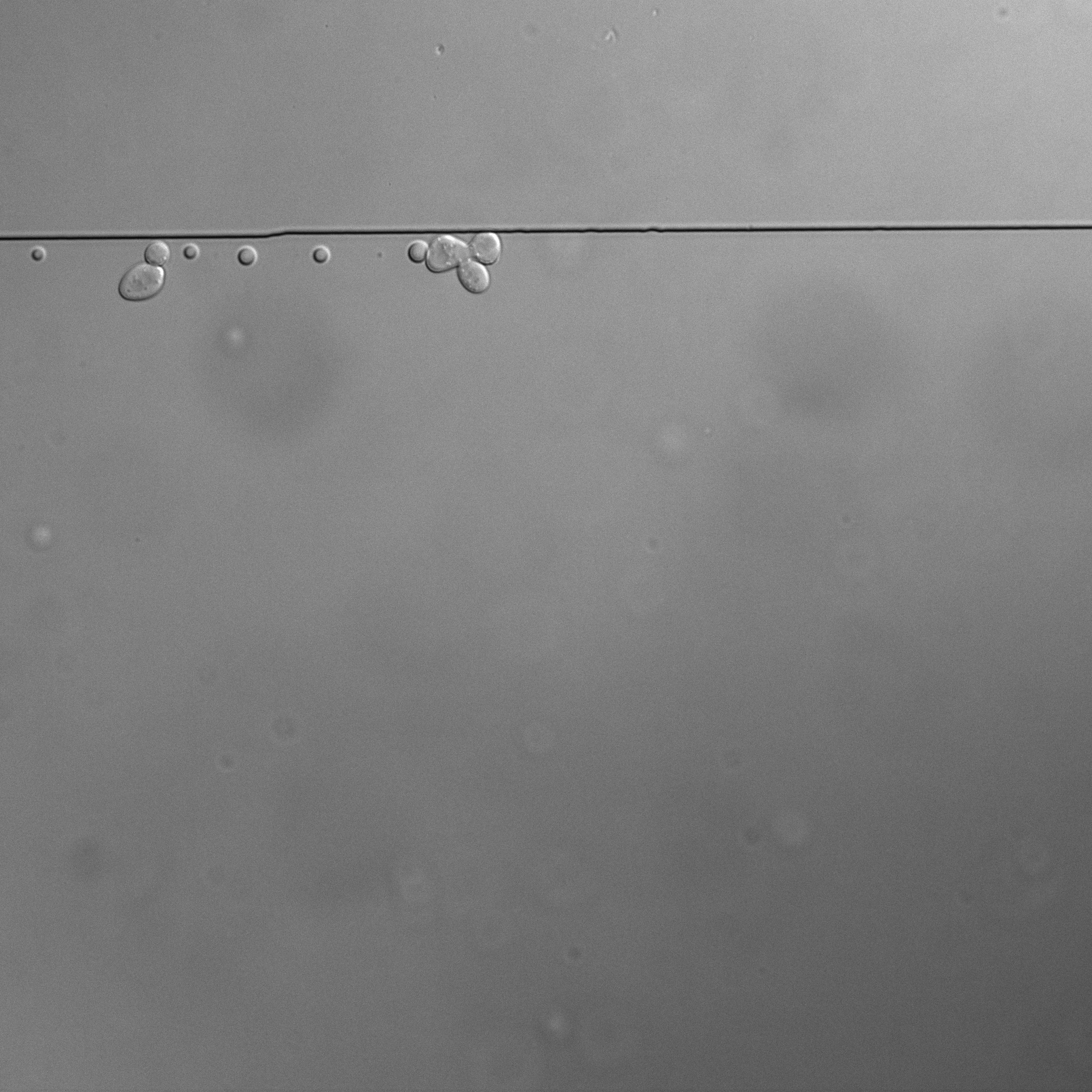

Supplement: Supplementary file 16 — Source data Fig. 3 [file 44318_2024_183_MOESM16_ESM.zip › Figure 3/3A/BF/060223_wt_ng_mk.cl1_series005_T032_BF_fr0.tif]

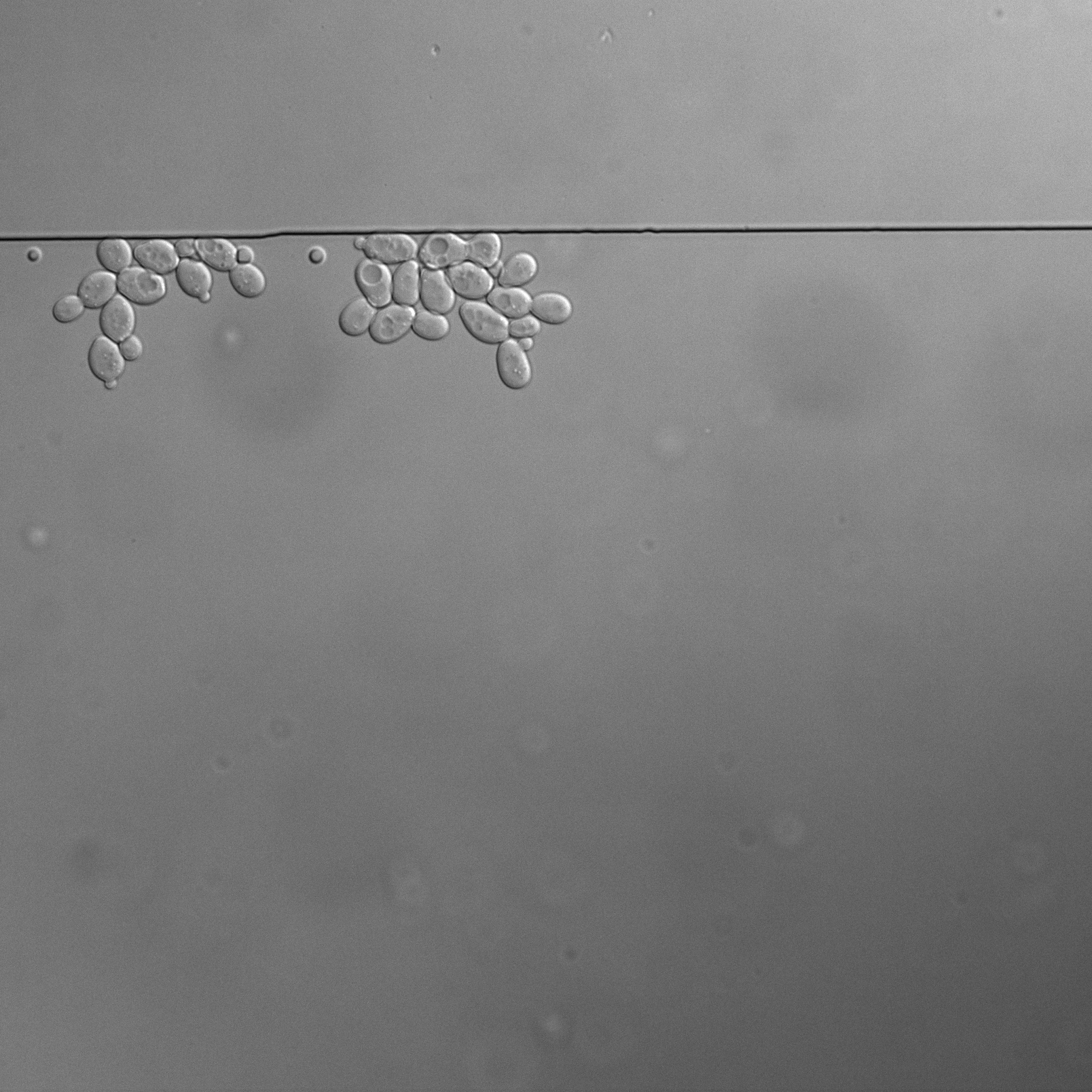

Supplement: Supplementary file 16 — Source data Fig. 3 [file 44318_2024_183_MOESM16_ESM.zip › Figure 3/3A/BF/060223_wt_ng_mk.cl1_series005_T032_BF_fr16.tif]

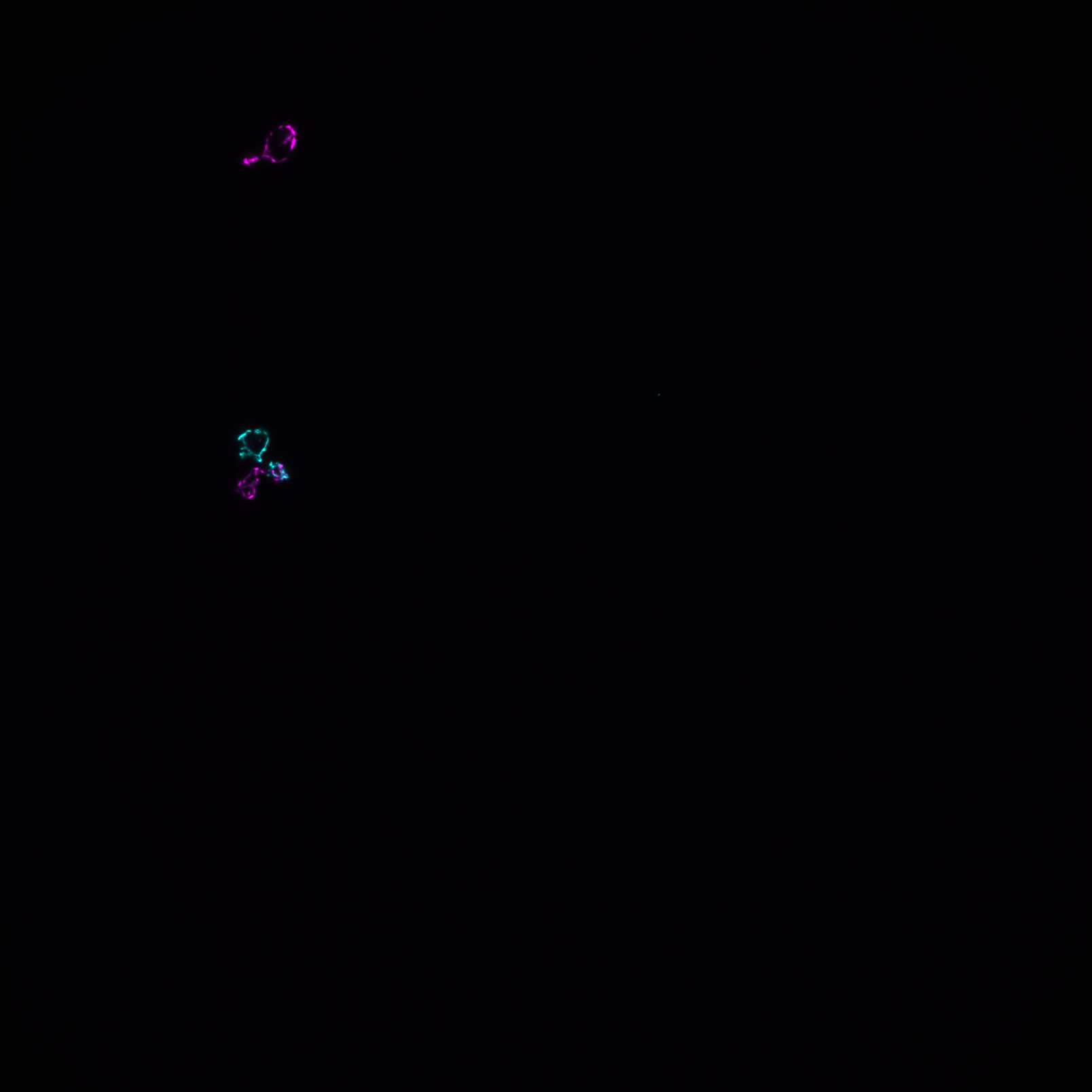

Supplement: Supplementary file 16 — Source data Fig. 3 [file 44318_2024_183_MOESM16_ESM.zip › Figure 3/3A/Merge/MAX_060223_wt_ng_mk.cl1_series005_T001_Composite_NG_mK_fr0.tif]

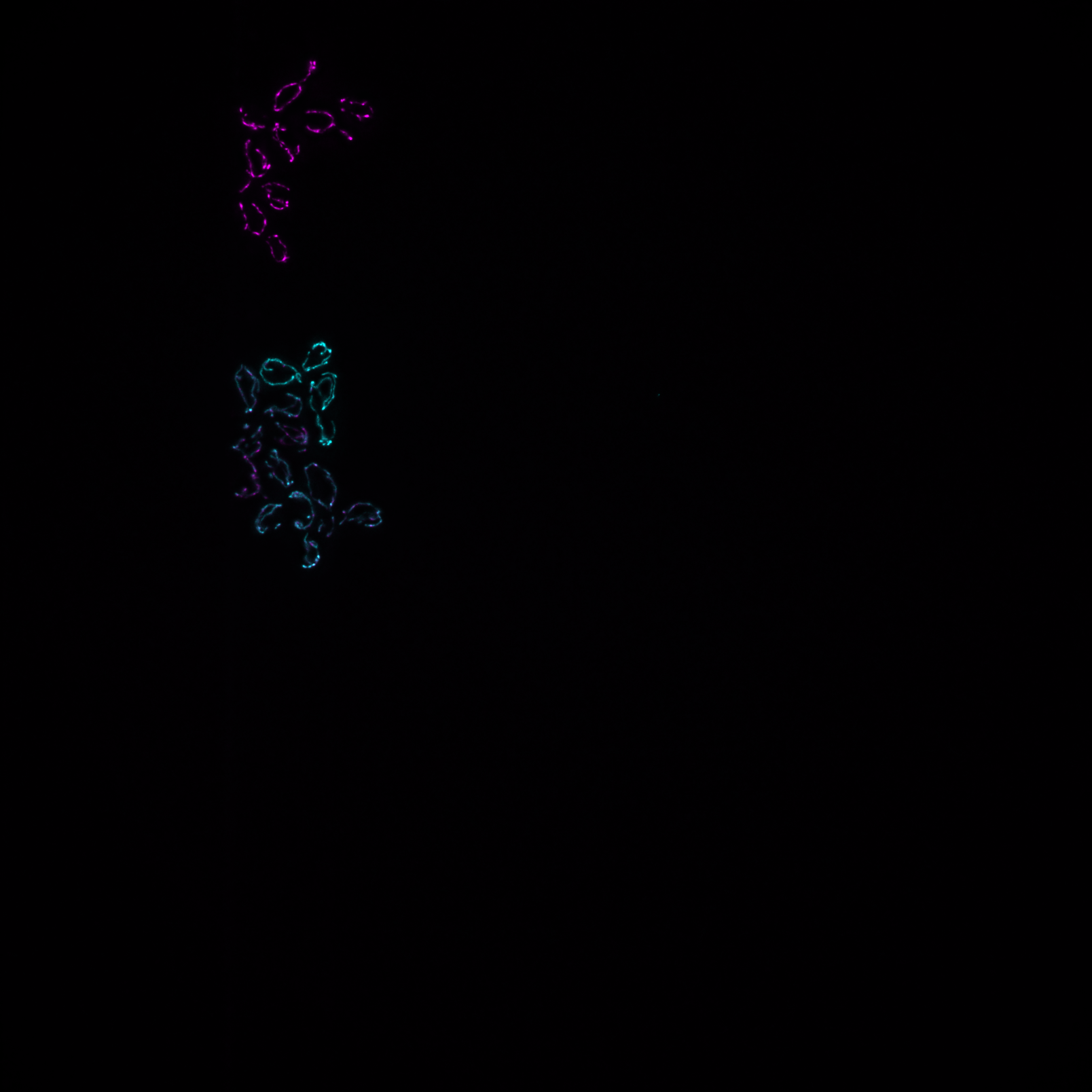

Supplement: Supplementary file 16 — Source data Fig. 3 [file 44318_2024_183_MOESM16_ESM.zip › Figure 3/3A/Merge/MAX_060223_wt_ng_mk.cl1_series005_T001_Composite_NG_mK_fr16.tif]

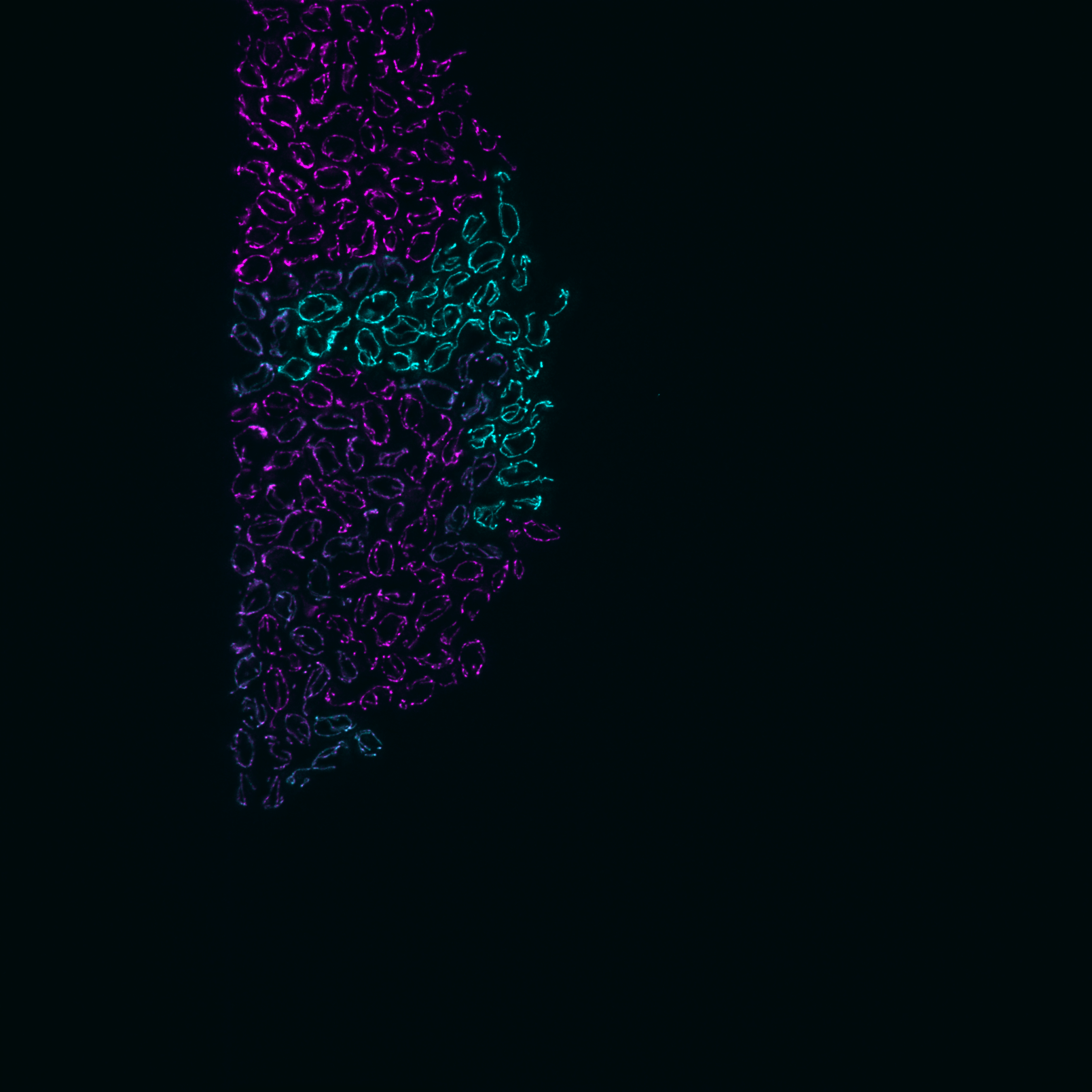

Supplement: Supplementary file 16 — Source data Fig. 3 [file 44318_2024_183_MOESM16_ESM.zip › Figure 3/3A/Merge/MAX_060223_wt_ng_mk.cl1_series005_T001_Composite_NG_mK_fr32.tif]

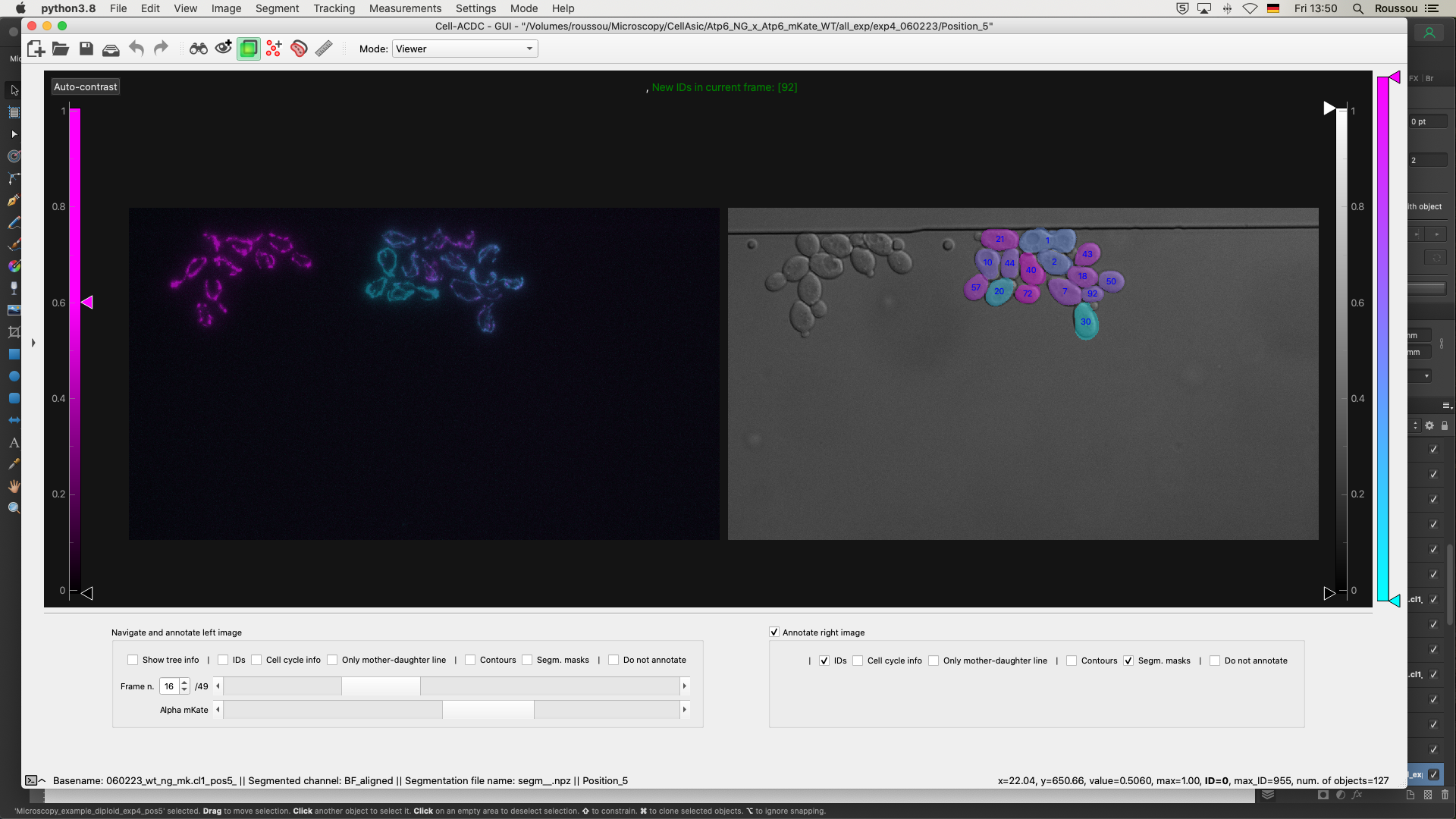

Supplement: Supplementary file 16 — Source data Fig. 3 [file 44318_2024_183_MOESM16_ESM.zip › Figure 3/3A/Masks/ACDC_wt_diploid_t4h.png]

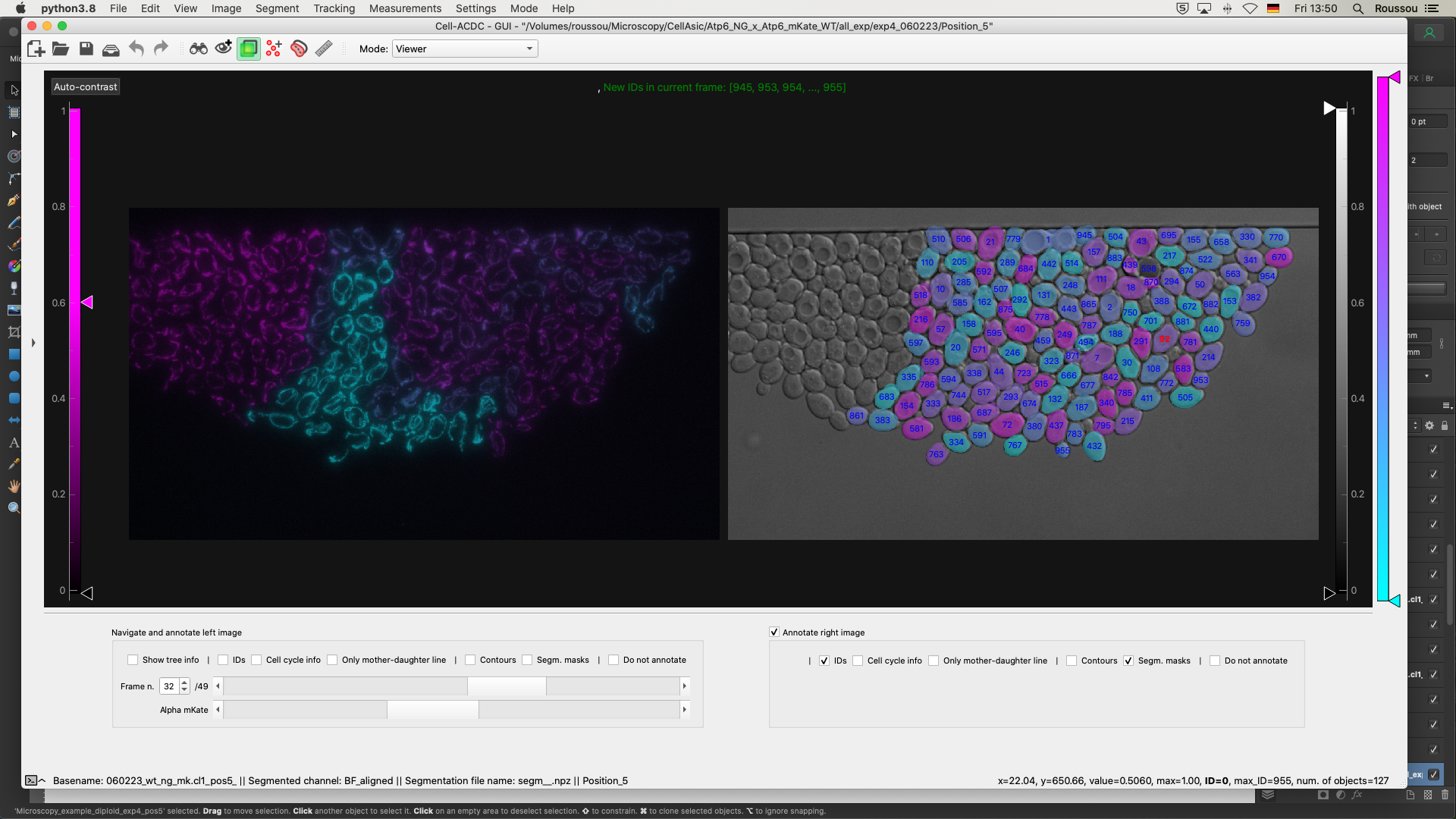

Supplement: Supplementary file 16 — Source data Fig. 3 [file 44318_2024_183_MOESM16_ESM.zip › Figure 3/3A/Masks/ACDC_wt_diploid_t8h.png]

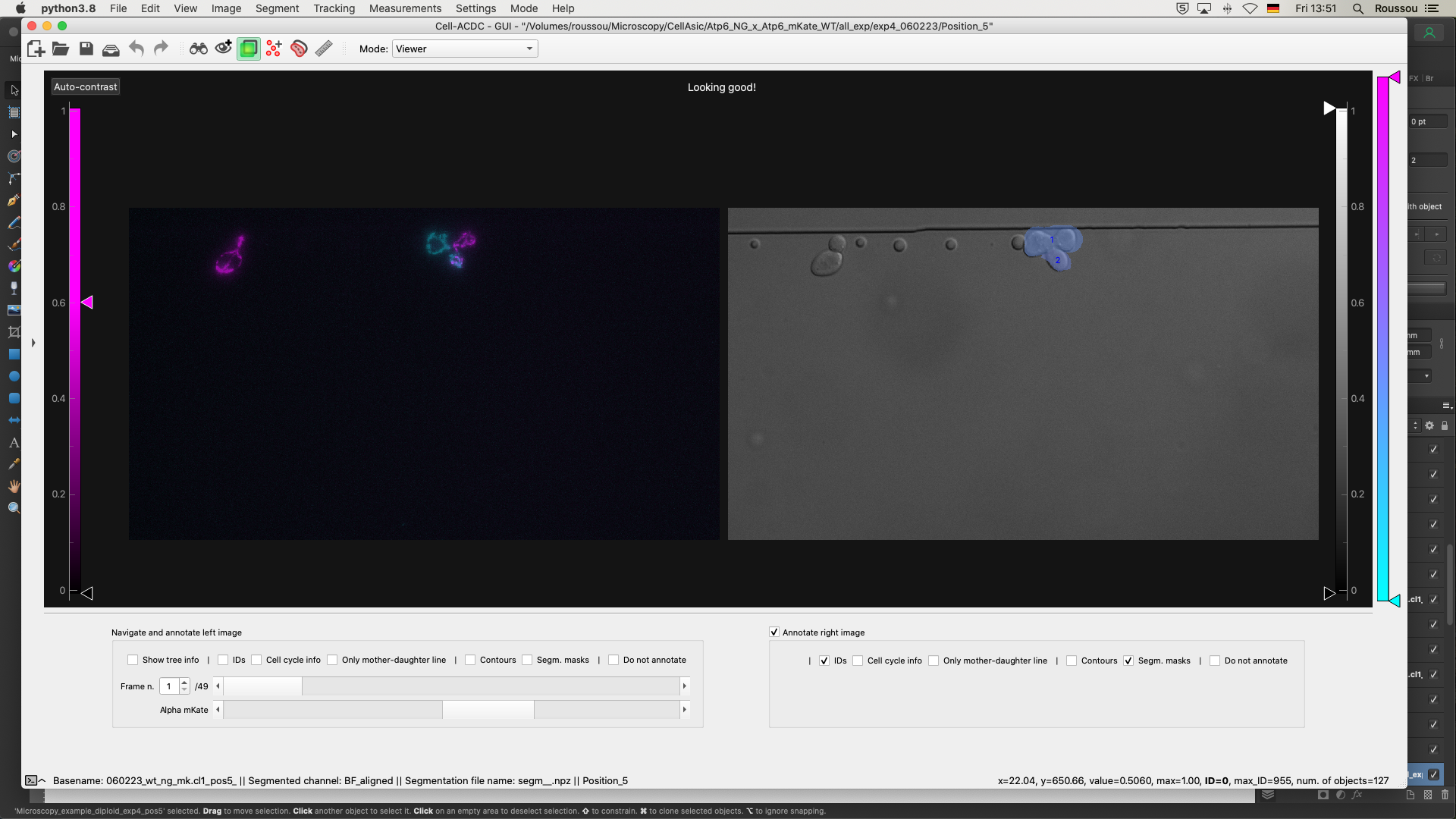

Supplement: Supplementary file 16 — Source data Fig. 3 [file 44318_2024_183_MOESM16_ESM.zip › Figure 3/3A/Masks/ACDC_wt_diploid_t0h.png]

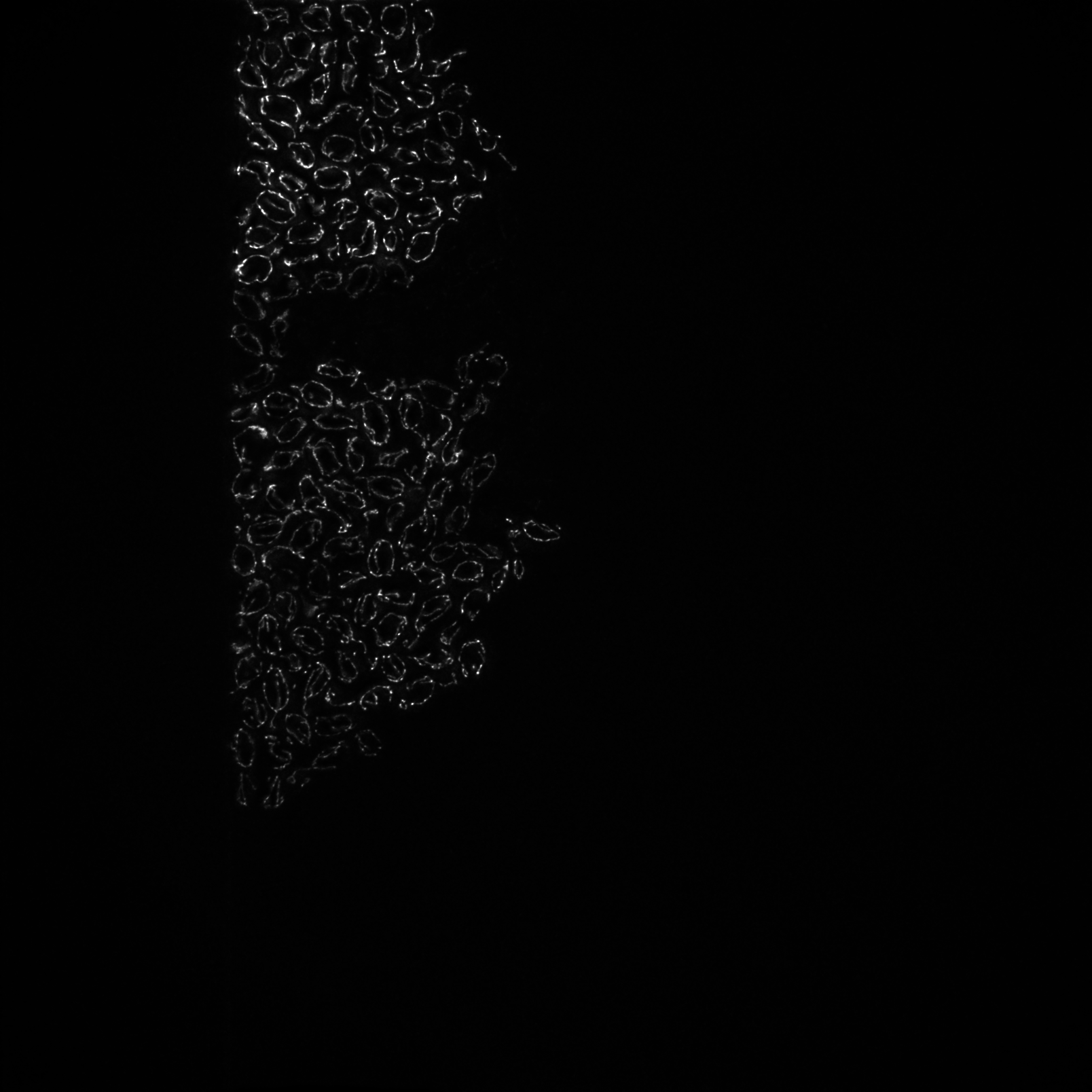

Supplement: Supplementary file 16 — Source data Fig. 3 [file 44318_2024_183_MOESM16_ESM.zip › Figure 3/3A/mKate2/MAX_060223_wt_ng_mk.cl1_series005_T032.ome_cmle_ch04_mKate_fr32.tif]

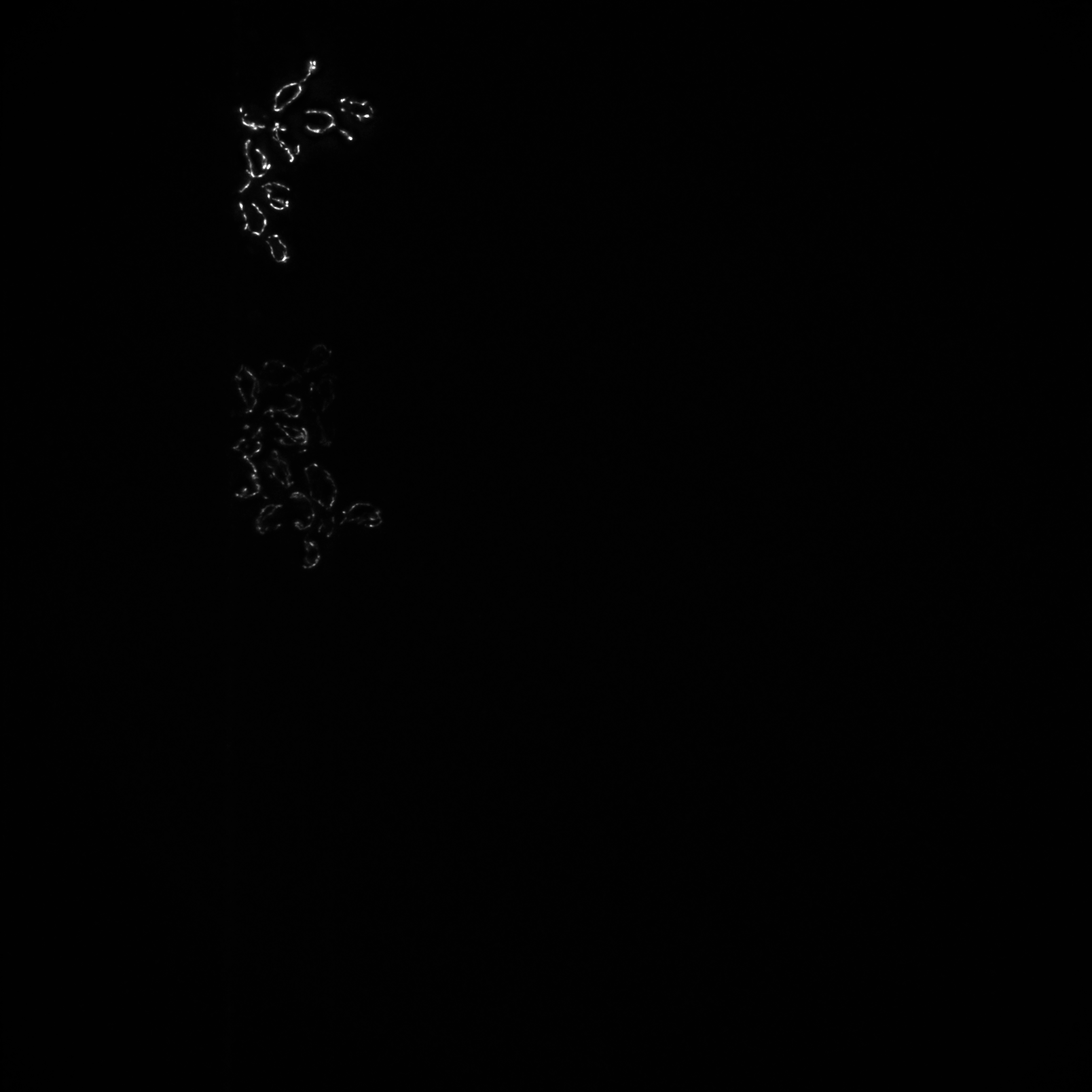

Supplement: Supplementary file 16 — Source data Fig. 3 [file 44318_2024_183_MOESM16_ESM.zip › Figure 3/3A/mKate2/MAX_060223_wt_ng_mk.cl1_series005_T016.ome_cmle_ch04_mKate_fr16_adjusted_hist.tif]

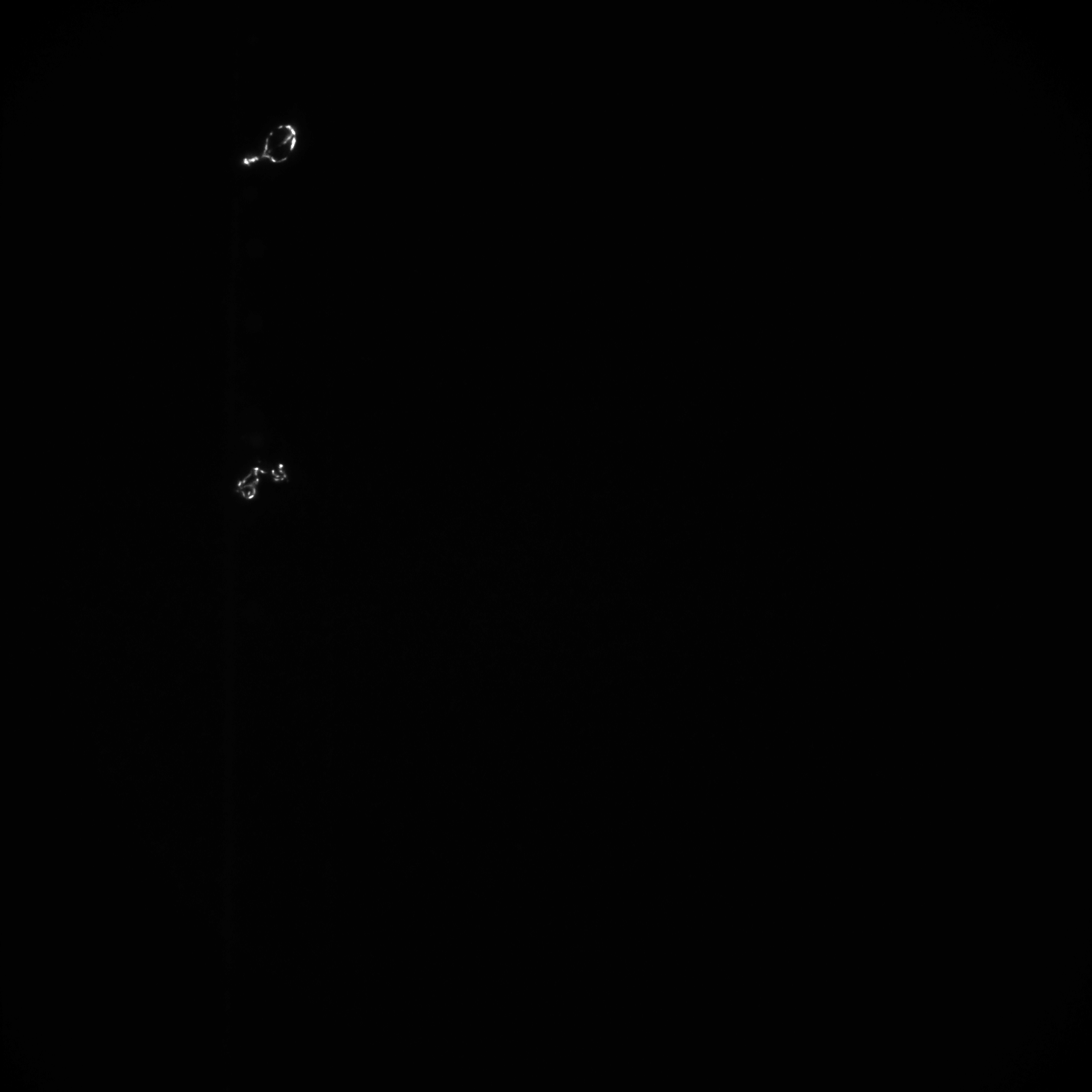

Supplement: Supplementary file 16 — Source data Fig. 3 [file 44318_2024_183_MOESM16_ESM.zip › Figure 3/3A/mKate2/MAX_060223_wt_ng_mk.cl1_series005_T001.ome_cmle_ch04_mKate_fr0.tif]

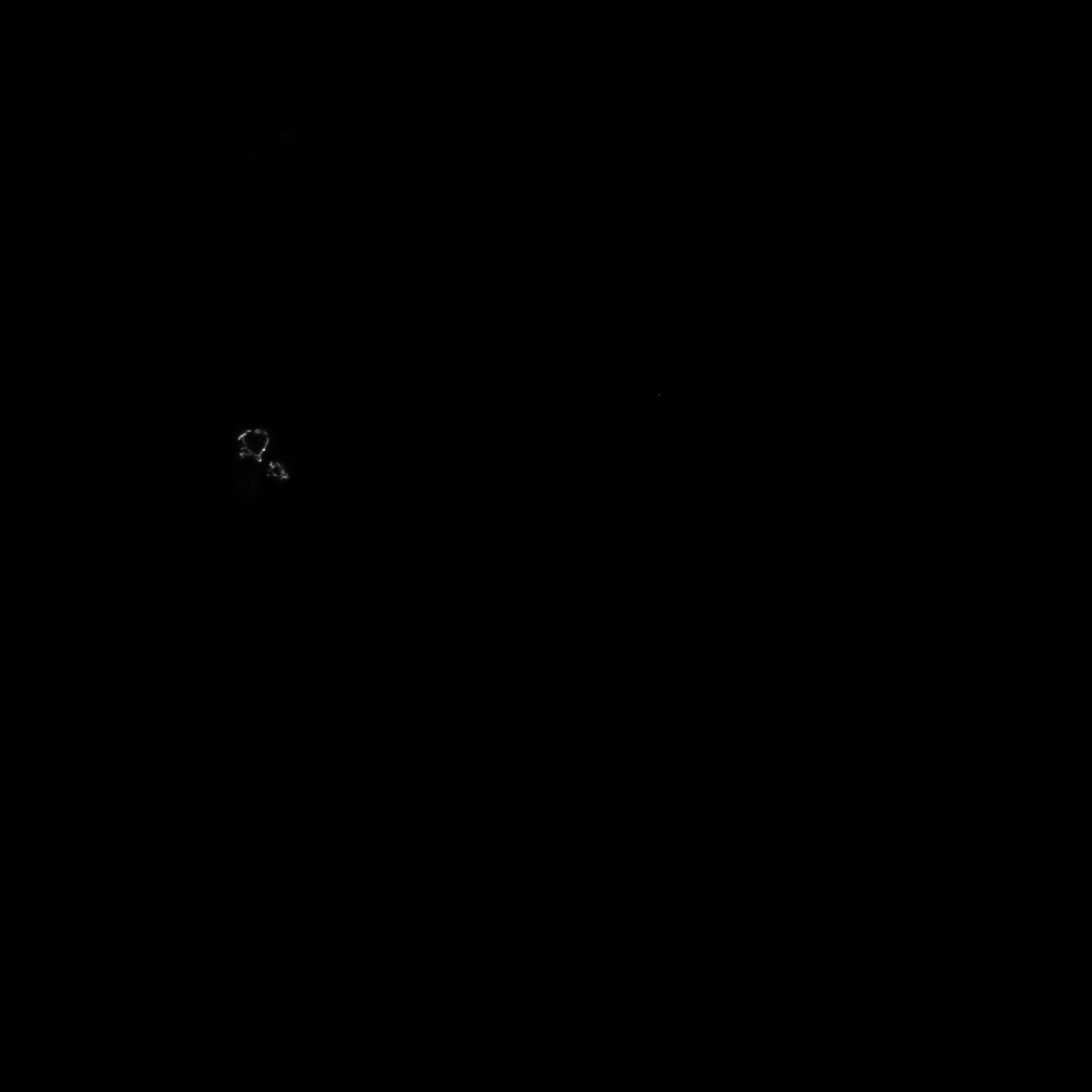

Supplement: Supplementary file 16 — Source data Fig. 3 [file 44318_2024_183_MOESM16_ESM.zip › Figure 3/3A/NG/MAX_060223_wt_ng_mk.cl1_series005_T001.ome_cmle_ch03_NG_fr0.tif]

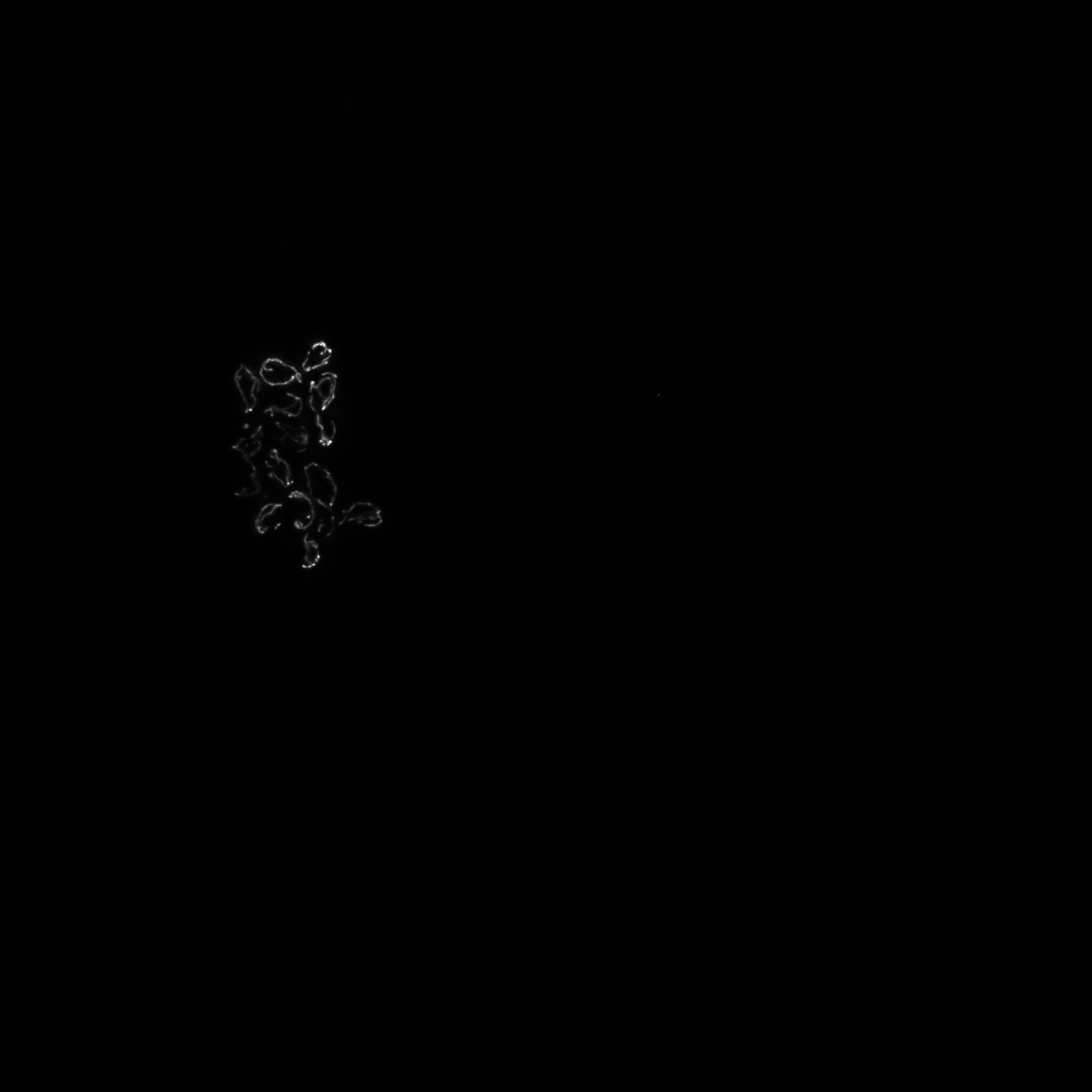

Supplement: Supplementary file 16 — Source data Fig. 3 [file 44318_2024_183_MOESM16_ESM.zip › Figure 3/3A/NG/MAX_060223_wt_ng_mk.cl1_series005_T016.ome_cmle_ch03_NG_fr16.tif]

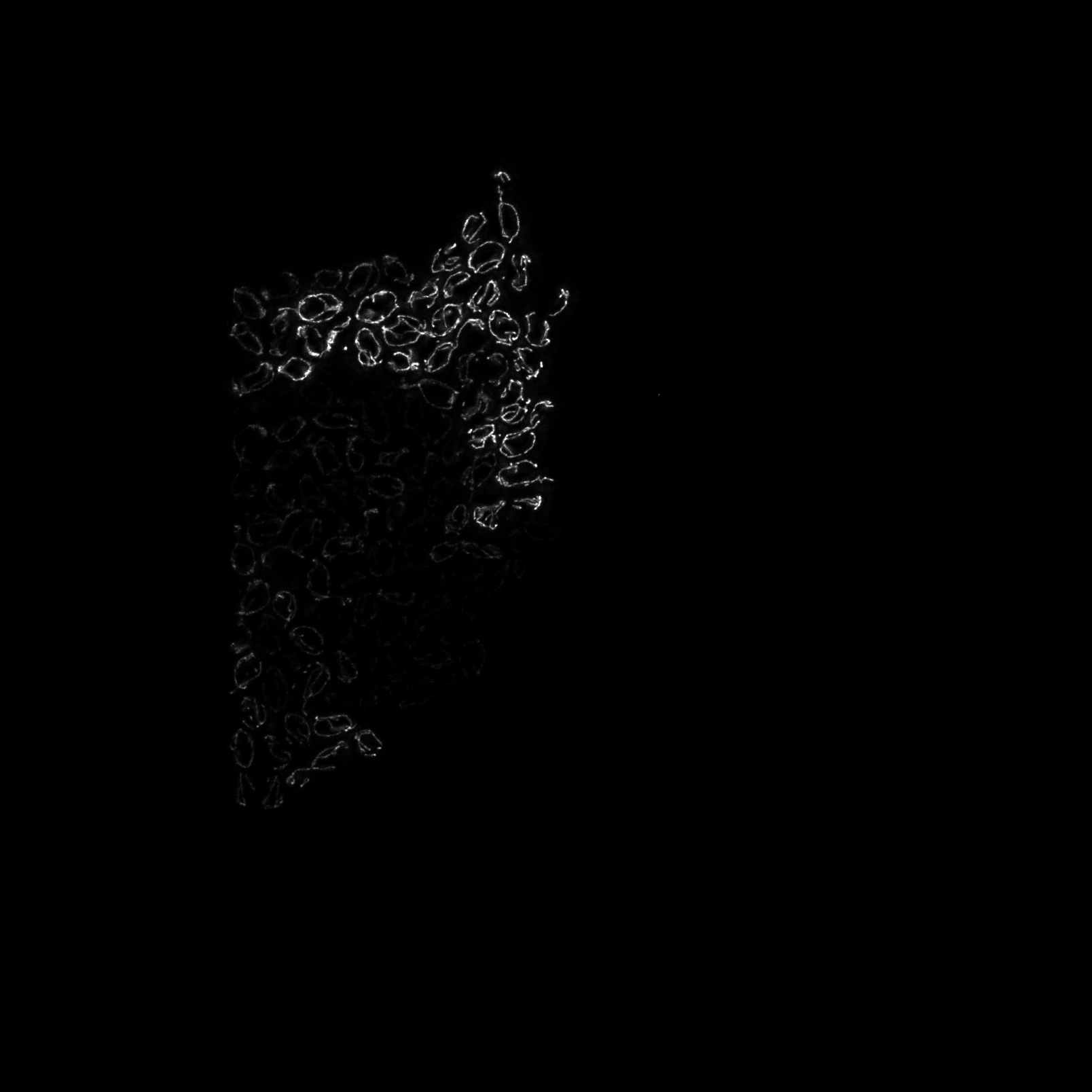

Supplement: Supplementary file 16 — Source data Fig. 3 [file 44318_2024_183_MOESM16_ESM.zip › Figure 3/3A/NG/MAX_060223_wt_ng_mk.cl1_series005_T032.ome_cmle_ch03_NG_fr32.tif]

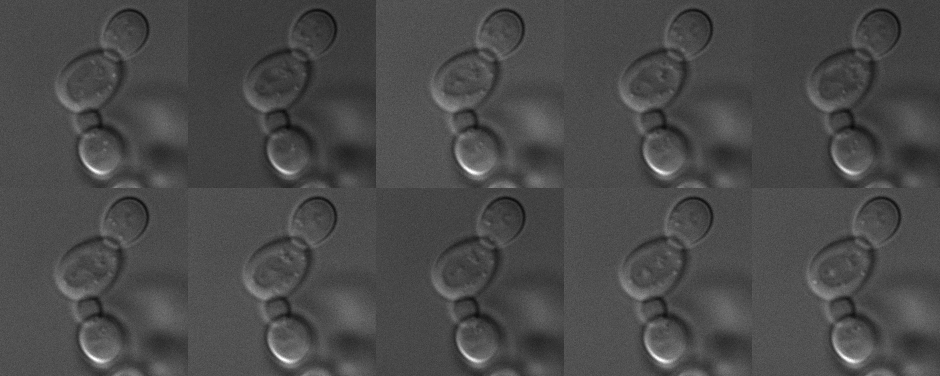

Supplement: Supplementary file 18 — Source data Fig. 5 [file 44318_2024_183_MOESM18_ESM.zip › Figure 5/5C/BF_cell1_montage.tif]

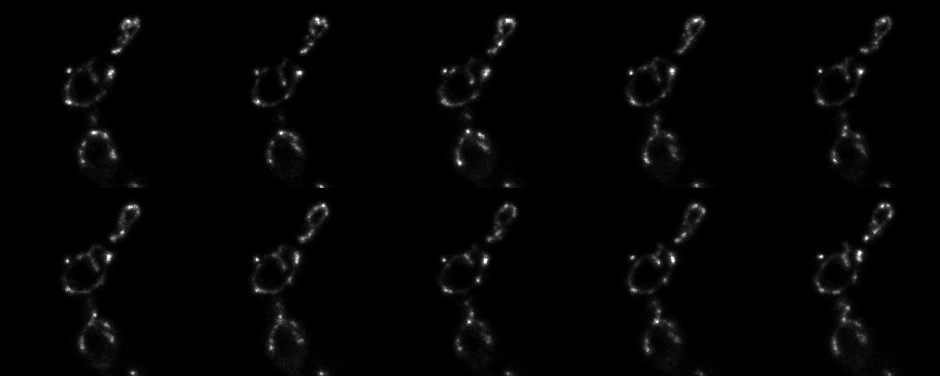

Supplement: Supplementary file 18 — Source data Fig. 5 [file 44318_2024_183_MOESM18_ESM.zip › Figure 5/5C/Laco_NG_cell1_montage.tif]

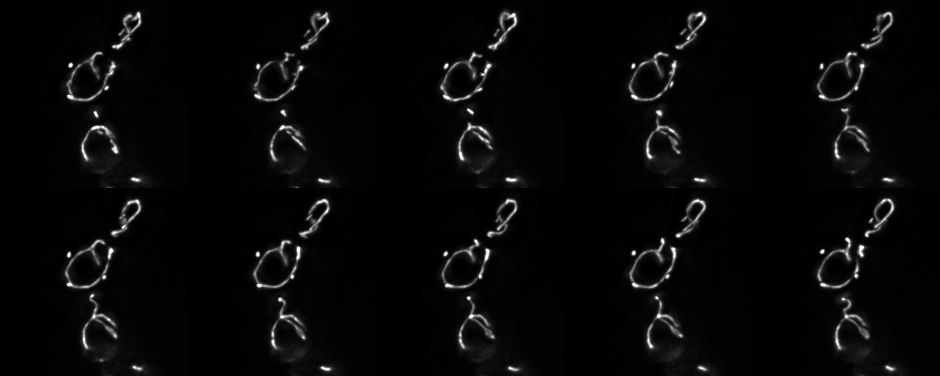

Supplement: Supplementary file 18 — Source data Fig. 5 [file 44318_2024_183_MOESM18_ESM.zip › Figure 5/5C/Matrix_mKate2_cell1_montage.tif]
