# Supplementary material for: Real-time assessment of mitochondrial DNA heteroplasmy dynamics at the single-cell level
Source: EMBO J. 2024 Aug 5;43(22):4. doi: 10.1038/s44318-024-00183-5 (PMC11574196; doi:10.1038/s44318-024-00183-5)
Supplement: Supplementary file 19 — Expanded View Figures [file 44318_2024_183_MOESM19_ESM.pdf]

Expanded View Figures

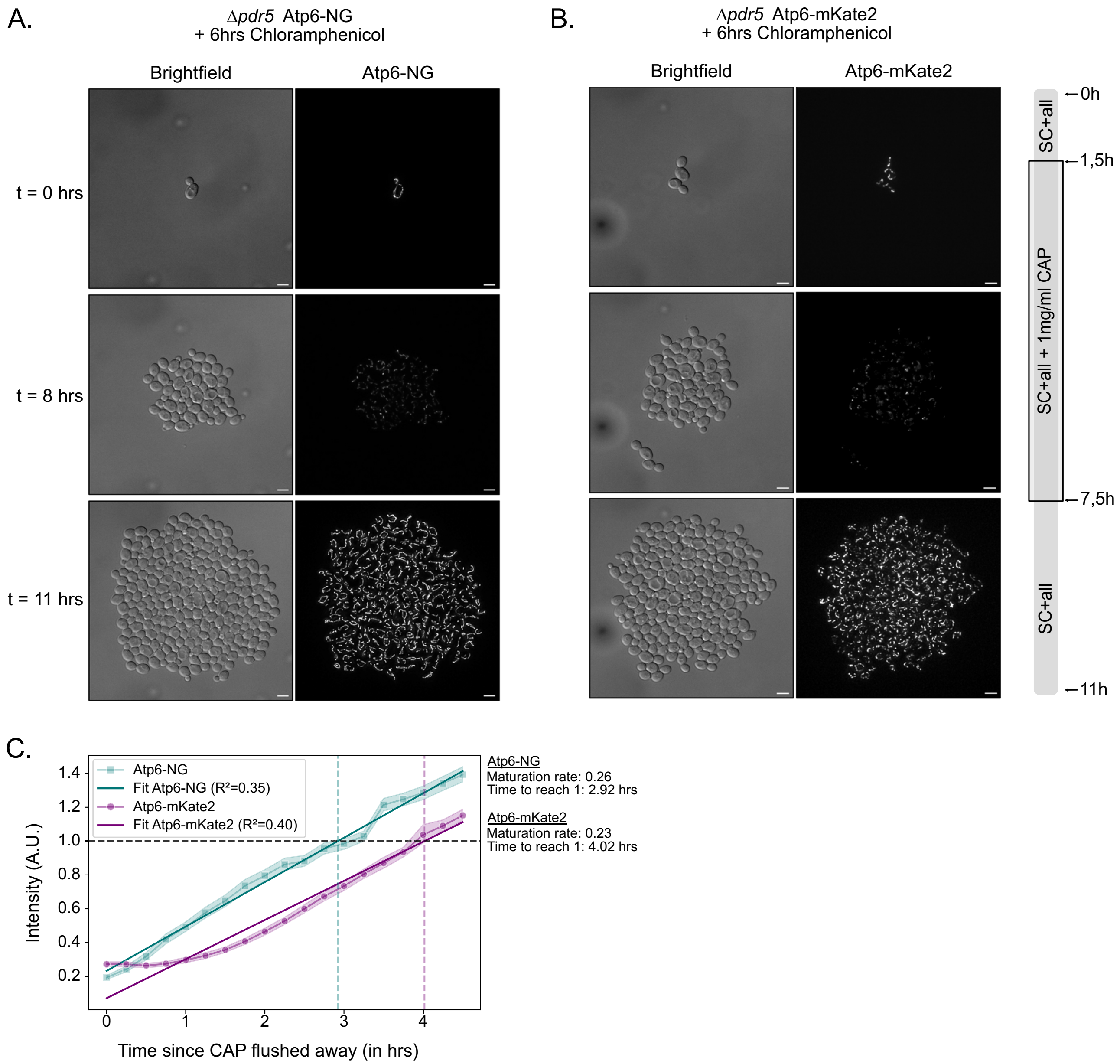

Figure EV1. mtDNA$^{Atp6\text{-}NG}$ and mtDNA$^{Atp6\text{-}mKate2}$ strains with temporary exposure to chloramphenicol.

(**A**, **B**) Yeast cells harboring mtDNA$^{Atp6\text{-}NG}$ (**A**) or mtDNA$^{Atp6\text{-}mKate2}$ (**B**) were imaged for 11 h ($N = 3$/strain). Chloramphenicol (1 mg/ml) was added after the first cell duplication, i.e., after 1.5 h of imaging, and was removed from the microfluidic chamber after 6 h of incubation. Fluorescence images are maximum-intensity projections of z-stacks, after deconvolution. Scale bars: 5 μm. (**C**) Line graph showing the fluorescent intensities of cells expressing Atp6-NG (cyan) or Atp6-mKate2 (magenta) upon washing off the Chloramphenicol (see Fig. 2F) after incubation with 1 mg/ml CAP for 6 h for sufficient mitochondrial translation inhibition. Curve fitting on the fluorescent intensity lines upon washing off the drug provides the maturation timings of the two Atp6 variants. Data represent the mean of all replicates per strain, and the shaded areas represent the 95% confidence interval. Fluorescent intensities were normalized to the cells at the first timepoint, for either of the fluorescent channels.

 © The Author(s)

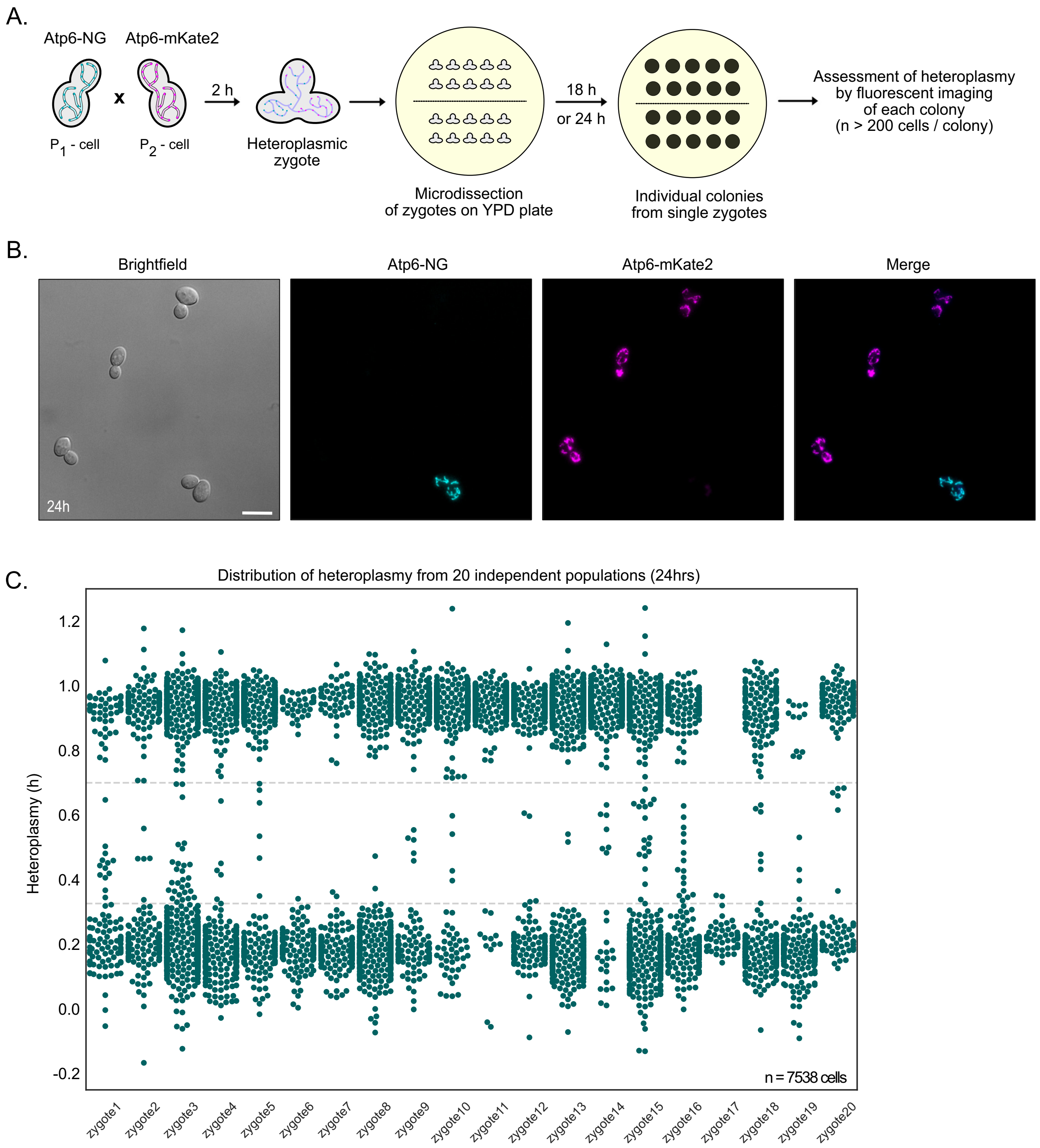

Figure EV2. 24-h heteroplasmy assessment from 20 independent populations.

(**A**) Schematic of the 24-h heteroplasmy assessment experiment. Cells harboring mtDNA$^{Atp6\text{-}NG}$ were mated with cells containing mtDNA$^{Atp6\text{-}mKate2}$ on a YPD plate. After a 2-h incubation, upon zygote formation, individual zygotes were micro-dissected and placed in different areas of a fresh YPD plate. Cells were kept for 24 h at 30 °C until colonies were formed. Cells from each colony, originating from a single zygote, were imaged to assess population homoplasmy levels, by calculating the proportion of cells exhibiting Atp6-NG and/or Atp6-mKate2 signal. (**B**) Representative image of a small field of view from one population, after 24 h. In total, 20 individual populations were screened, each originating from a diploid heteroplasmic zygote. Cells harboring mtDNA$^{Atp6\text{-}NG}$ are shown in cyan, while cells having kept the mtDNA$^{Atp6\text{-}mKate2}$ are depicted in magenta. Fluorescence images are maximum-intensity projections of z-stacks, after deconvolution. Scale bars: 5 µm. (**C**) Heteroplasmy distributions from all 20 independent diploid populations after 24 h of growth on the YPD plate ($N = 20$ zygotes/$n > 200$ cells/colony). Each dot represents a single cell. Heteroplasmy values below 0.33 or above 0.71, the homoplasmy thresholds, represent cells harboring mainly mtDNA$^{Atp6\text{-}NG}$ or mtDNA$^{Atp6\text{-}mKate2}$, respectively. As apparent from the graph, some cells for each population still exhibit a heteroplasmic state, which is evident from a h-value between 0.33 and 0.71.

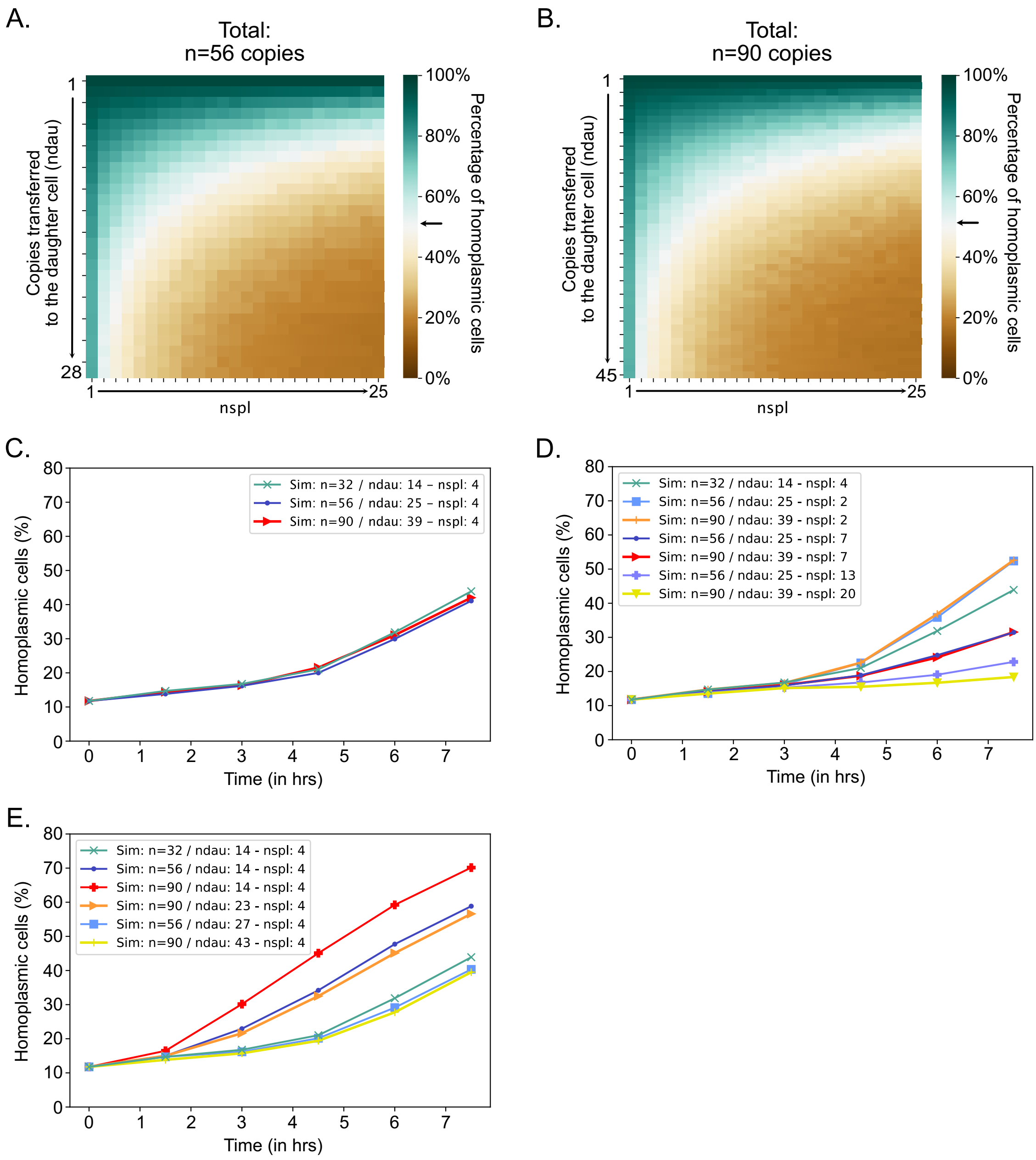

Figure EV3. Simulations of homoplasmy establishment in cell populations with increased mtDNA copy number.

(**A**) Heatmap showing the percentage of homoplasmic cells at timepoint $t = 7.5$ h, for different *ndau* and *nspl* pairs, for $n = 56$ mtDNA copies. The maximum value of *ndau* is defined by the half ($n = 28$) of the total mtDNA copies in the founder cell ($n = 56$). The arrow indicates the proportion (50%) of cells being virtually homoplasmic in the empirical data, based on the pre-established homoplasmy cutoffs. Each simulation with any given combination of the two parameters has been run ten times. (**B**) Heatmap showing the percentage of homoplasmic cells at timepoint $t = 7.5$ h, for different *ndau* and *nspl* pairs, for $n = 90$ mtDNA copies. The maximum value of *ndau* is defined by half ($n = 45$) of the total mtDNA copies in the founder cell ($n = 90$). The arrow indicates the proportion (50%) of cells being virtually homoplasmic in the empirical data, based on the pre-established homoplasmy cutoffs. Each simulation with any given combination of the two parameters has been run ten times. (**C**) Percentage of homoplasmic cells in simulated cell populations with $n = 32$ copies, relative to populations with $n = 56$ copies or $n = 90$ copies/cell. The number of copies transferred to the daughter cell (*ndau*) has been kept proportional to the total copy number in each of the simulations, that is 44\% of the total copies gets transferred to the daughter cell. The parameter *nspl* has been kept identical. (**D**) Percentage of homoplasmic cells in simulated cell populations with $n = 32$ copies, relative to populations with $n = 56$ copies or $n = 90$ copies/cell, with varying *nspl* values. The *ndau* parameter is proportional to the total copy number (44%), while the *nspl* parameter varies from low to high values per simulation. A comparison of these curves demonstrates the effect of lower or higher fission-fusion frequencies on the speed of segregation. The combination of $ndau = 14$ and $nspl = 4$ in cells with $n = 32$ copies is used as a point of reference, across all plots. (**E**) Percentage of homoplasmic cells in simulated cell populations with $n = 32$ copies, relative to populations with $n = 56$ copies or $n = 90$ copies/cell, with identical *nspl* values, and different levels of *ndau*. Comparison of these curves demonstrates the effect of lower or higher numbers of mtDNA copies being transferred to the daughter cells on the speed of segregation. The combination of $ndau = 14$ and $nspl = 4$ in cells with $n = 32$ copies is used as a point of reference, across all plots.

 © The Author(s)

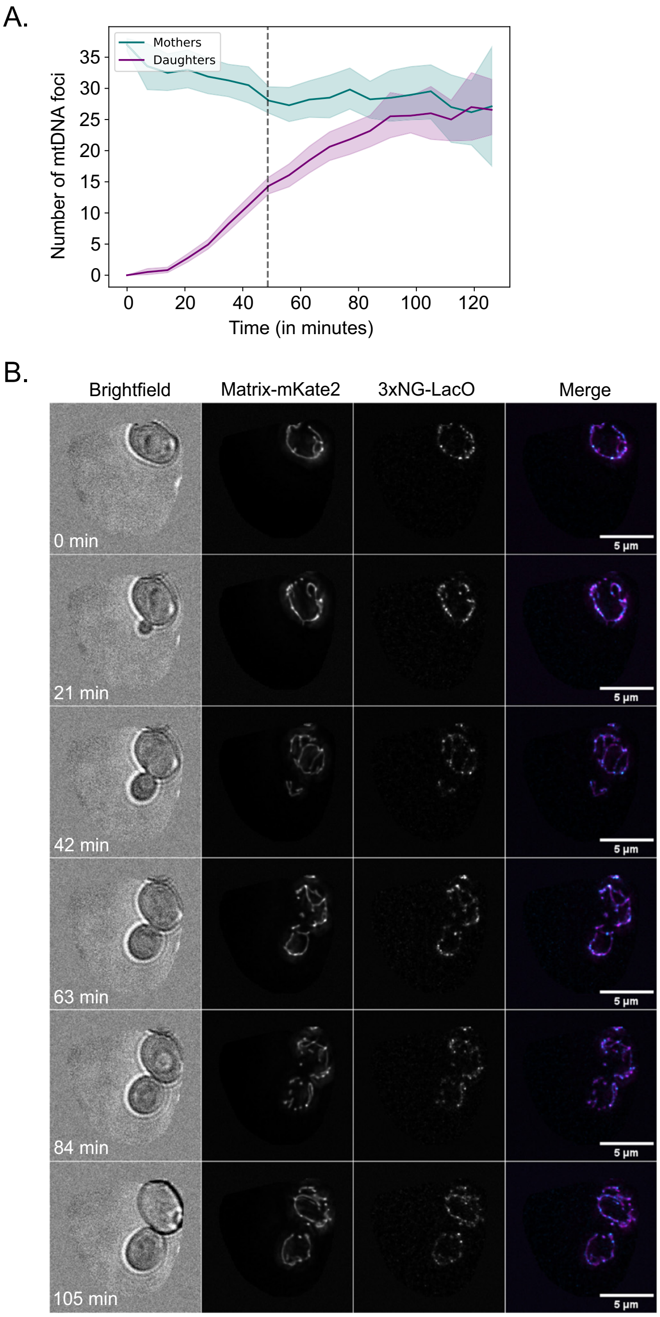

Figure EV4. Number of mtDNA foci in mother-daughter pairs during a complete cell cycle.

(**A**) Diploid cells expressing mitochondrially targeted 3xNG-LacI and mKate2 were harvested at the log phase and imaged for 2 h. The number of mtDNA foci were counted in virgin mothers (M) and their emerging daughters (D) ($n = 59$ M-D pairs). The dashed line represents the average period of time ($t = 47.5$ min) that mitochondrial content was exchanged between mothers and daughters, as shown in Fig. 5D. (**B**) Example time-lapse images of a virgin mother and its emerging bud. All cells express mitochondrially targeted 3xNG-LacI, used for visualizing the LacO-mtDNA foci, and mitochondrially targeted mKate2 to visualize the mitochondrial network. Mother-daughter pairs were cropped manually prior to segmentation and analysis.

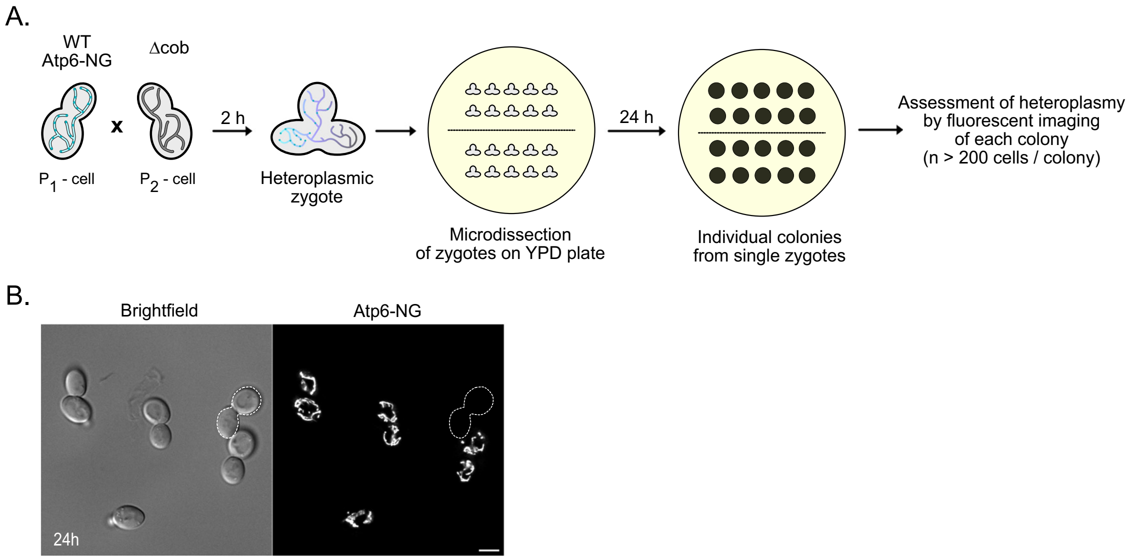

Figure EV5. 24-hr heteroplasmy assessment in colonies with competing mtDNA qualities.

(**A**) Schematic of the 24 h heteroplasmy assessment experiment of matings between cells harboring intact or mutant mtDNA. Cells harboring intron-containing $mtDNA^{Atp6\text{-}NG}$ were mated with cells containing intronless $mtDNA^{il\text{-}\Delta cob}$ on a YPD plate. After a 2 h incubation, upon zygote formation, individual zygotes were micro-dissected and placed in different areas of a fresh YPD plate. Cells were kept for 24 h at 30 °C until colonies had formed. Cells from each colony, originating from a single zygote, were imaged to assess the percentage of cells with fluorescent ($mtDNA^{Atp6\text{-}NG}$) or 'dark' (*$mtDNA^{il\text{-}\Delta cob}$*) mitochondria. (**B**) Representative image of a small field-of-view from one population, after 24 h. Diploid cells harboring $mtDNA^{Atp6\text{-}NG}$ are exhibiting fluorescence, while 'dark' cells (indicated by a white outline) do not contain $mtDNA^{Atp6\text{-}NG}$. Fluorescence images are maximum-intensity projections of z-stacks, after deconvolution. Scale bars: 5 µm.

 © The Author(s)
